# Effects of Emissions From Oriented Strand Board on the Development of Atopic Dermatitis Using Two Different Experimental Mouse Models

**DOI:** 10.1111/exd.70086

**Published:** 2025-03-20

**Authors:** Evelyn Schneider, Katja Butter, Benjamin Schnautz, Stephanie Musiol, Johanna Grosch, Sonja Schindela, Manuel Garcia‐Käufer, Richard Gminski, Stefan Haak, Martin Ohlmeyer, Carsten B. Schmidt‐Weber, Stefanie Eyerich, Julia Esser‐von Bieren, Francesca Alessandrini

**Affiliations:** ^1^ Center of Allergy and Environment (ZAUM), Technical University of Munich (TUM) and Helmholtz Zentrum München German Research Center for Environmental Health Neuherberg Germany; ^2^ Thünen Institute of Wood Research Hamburg Germany; ^3^ Institute for Infection Prevention and Control, Medical Center ‐ University of Freiburg, Faculty of Medicine University of Freiburg Freiburg Germany; ^4^ Member of the German Center of Lung Research (DZL) Munich Germany

**Keywords:** atopic dermatitis, atopic march, inflammation, lipid mediators, mouse models, oriented strand board, volatile organic compounds

## Abstract

Atopic dermatitis (AD) is an allergic skin disease widespread in children, which later in life can predispose them to asthma. Oriented strand board (OSB), increasingly used in the construction industry, emits volatile organic compounds in the indoor air, some of which may exacerbate ad development in humans. The aim of this study was to evaluate the effects of OSB emissions on the development of AD and lung inflammation. Two different murine ad models, induced by calcipotriol or oxazolone, were exposed to higher‐ or lower‐emitting OSB throughout the experiments. Physiological, biochemical, and immunological parameters of skin disease development, as well as lung inflammatory parameters, were evaluated. Exposure to higher‐emitting OSB, characterised especially by high 3‐carene emissions, exacerbated some parameters of ad, such as skin barrier function and thickness, with accumulation of eosinophils and 15‐lipoxygenase (15‐LOX)‐driven mediators in both models, whereas IL‐4 or 5‐LOX‐positive cells were increased in only the calcipotriol or oxazolone model, respectively. In the lungs of calcipotriol‐treated mice, higher‐emitting OSB increased lung eosinophil recruitment. Exposure to lower‐emitting OSB had no or even beneficial effects on the skin or lungs of murine ad models. 3‐carene in OSB emissions, alone or in combination with other substances, may promote the development of ad and prime the lungs towards an allergic phenotype. Identification and quantification of potentially harmful emitting sources in indoor air may be important for ad prevention or control.

## Graphical abstract

Higher‐emitting OSB exacerbates the development of atopic dermatitis and increases lung eosinophil recruitment. Lower‐emitting OSB has no or even beneficial effects in both atopic skin and lungs. Direct VOC exposures from OSB in mouse in vivo and human in vitro models

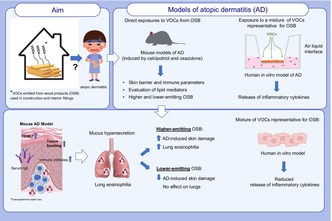

**Abbreviations**
15(S)‐HETE: 15(s)‐hydroxy eicosatetraenoic acid
ad
: atopic dermatitis
ALI: air‐liquid interface
COX: cyclooxygenase
EtOH: ethanol
LOX: lipoxygenase
LTs: leukotrienes
OSB: oriented strand board
PG: prostaglandin
TCS: T cell supernatant
TEWL: Transepidermal water loss
TVOCs: total volatile organic compounds
VOC: volatile organic compound

## 1 Introduction

Based on our living behaviours and work habits, we spend most of the day indoors. Due to the increasing airtightness of energy‐efficient constructions, total volatile organic compounds (TVOCs) released into the interior space from various sources [1, 2, 3, 4] accumulate in indoor air. Oriented strand board (OSB) is frequently used in the interior of houses as sheathing in walls, flooring, and roof decking or for furniture. Because in Europe OSB is primarily made from pinewood, it is associated with typical volatile emissions at elevated concentrations [5]. OSB emissions consist mainly of terpenes, especially α‐pinene and 3‐carene, and to a lower extent of aldehydes, especially hexanal, and only to a small percentage of organic acids [6]. While terpene emissions decrease over time relatively quickly, especially in the first couple of months, emissions of aldehydes tend to first increase after production due to auto‐oxidation of fatty acids on the board's surface and decrease later on [6, 7, 8, 9].

Atopic dermatitis (ad) is an inflammatory skin disease affecting 15%–20% of children [10] and 1%–3% of adults worldwide [11]. It usually starts in early childhood and it has been associated with a predisposition to develop other allergic diseases like allergic asthma later in life, in the context of the so‐called atopic march [12, 13]. The pathogenesis of ad involves a complex interaction between genetic, immunologic, and environmental factors, leading to a type 2‐dominated immunity accompanied by an impaired skin barrier, inflammation, and elevated serum IgE levels [14, 15, 16]. Eicosanoids, such as prostanoids and leukotrienes (LTs), bioactive lipid mediators derived from arachidonic acid by the activity of cyclooxygenase (COX) and lipoxygenase (LOX) pathways, also represent an important layer of immune regulation in type 2‐dominated disorders [17]. In the context of inflammatory skin disorders, various eicosanoids, including prostaglandin (PG) E2 and LTB_4_, have been detected in lesional skin, supporting their critical roles in the development of skin inflammation [18, 19].

The impact of indoor VOCs on allergic diseases is a matter of growing interest. Whilst wood‐ or OSB‐related VOCs exhibit beneficial effects on respiratory allergy [20, 21], several epidemiological studies have reported associations between various indoor ambient VOCs linked for example to renovation activities and atopic dermatitis in children [22, 23, 24, 25, 26]. In addition, a clinical research study demonstrated that a mixture of VOCs increased the susceptibility of ad skin to allergen exposure [27]. To which extent the risk associated with mixtures of VOCs on ad can be applied to the widely used OSB has not been investigated so far. For this purpose, we analysed ad development in two different murine ad models combined with a direct exposure to higher and lower OSB emissions. We demonstrate that higher‐emitting OSB enhances key inflammatory parameters and thus may promote the development of ad with a potential impact on the atopic march, whereas lower‐emitting OSB displays no, or rather beneficial effects.

## 2 Materials and Methods

### 2.1 Experimental Models of AD and Exposure to OSB Emissions

Two different murine models of AD (calcipotriol‐ and oxazolone‐induced), well characterised in [28] were exposed to higher‐ or lower‐emitting OSB, and physiological, biochemical, and/or immunological parameters of disease progression were recorded in the skin and lungs. Experiments were carried out under federal guidelines for the use and care of laboratory animals and approved by the government of the district of upper Bavaria (Approval n. ROB‐55.2‐2532.VET_02‐16‐198). To translate our findings to humans, a human in vitro model of ad (Approval n. 5590/12 and 44/16 S, Technical University of Munich) was exposed to a VOCs mixture simulating OSB emissions, and cytokine release was evaluated. For all methodological details, see the online Supporting Information.

### 2.2 Statistics

Graphical representations and statistics were done by Prism 7.0 (GraphPad Software, La Jolla, CA, USA). VOCs from higher‐ and lower‐emitting OSB were compared by multiple *t*‐tests with the Holm–Sidak method. Body weight, TEWL, pH, ear swelling, and IgE were analysed by two‐way analysis of variance (ANOVA) and all other data by one‐way ANOVA with Bonferroni post hoc test. Data were presented as boxplots or mean ± SD. *p* < 0.05 was considered significant.

## 3 Results

### 3.1 Long‐Term Storage of OSB Decreases VOC Emissions

**FIGURE 1:**
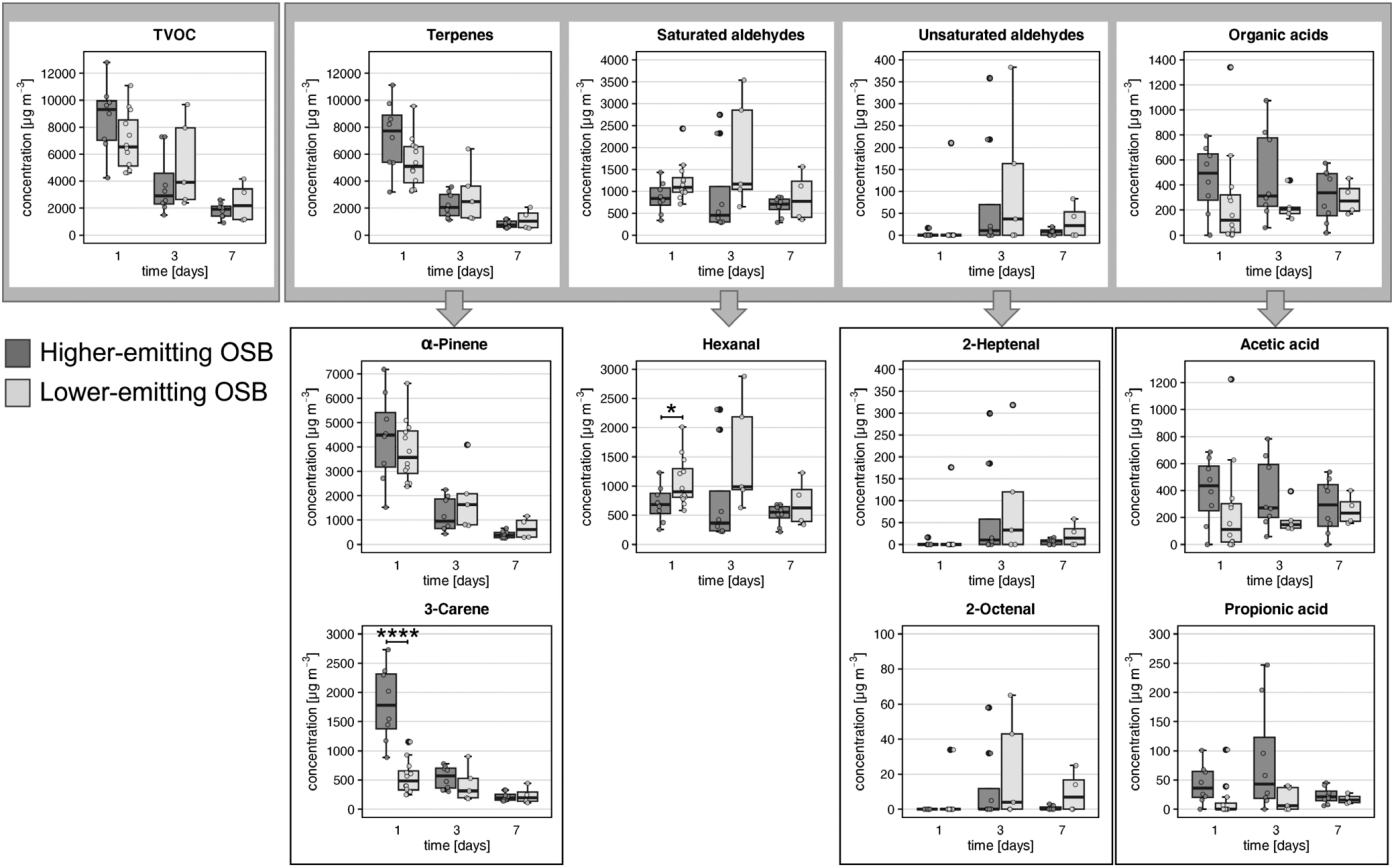
TVOC and representative VOC monitoring of mouse cages. TVOC monitoring was carried out 3 times a week for higher‐ and lower‐emitting OSB, which belonged to the same batch of the higher‐emitting OSB, but were stored for 1 year at −20°C (dark and light grey bars, respectively). Cumulative TVOC concentrations, and subsequent main representatives: Terpenes, aldehydes and organic acids are depicted. Data represent pooled concentrations of compounds measured in mouse cages during each week of exposure for the whole duration of the experiment. Dark bars, day 1, day 3 and day 7: *N* = 8. Light bars, day 1: *N* = 12, day 3: *N* = 5, day 7: *N* = 4. Boxplots depict minimum, 25th percentile, median, 75th percentile, maximum and outliers. *p < 0.05, ****p < 0.0001, multiple *t*‐test with Holm‐Sidak method.

Higher‐emitting OSB was characterised by high TVOC concentrations measured in mouse cages [median: 9314 μg/m^3^ (day 1), 1905 μg/m^3^ (day 7)], (Figure 1, dark bars). In particular, terpene concentrations were high on day 1 (7725 μg/m^3^), with α‐pinene reaching a median of 4488 μg/m^3^ and 3‐carene of 1782 μg/m^3^. By day 7, both substances decreased up to 10% of the initial concentration (α‐pinene, 378 μg/m^3^; 3‐carene, 195 μg/m^3^). Contrarily, saturated aldehydes, mainly hexanal, remained constant over the exposure period [836 μg/m^3^ (day 1), 706 μg/m^3^ (day 7)] and unsaturated aldehydes, mainly 2‐heptenal and 2‐octenal, were lower compared to saturated aldehydes [0 μg/m^3^ (day 1), 8 μg/m^3^ (day 7)]. After 1 year‐long storage at −20°C (lower‐emitting OSB, Figure 1, light bars), moderately lower TVOC concentrations were measured on day 1 (median: 6547 μg/m^3^), slightly decreasing later on [3912 μg/m^3^ (day 3), 2174 μg/m^3^ (day 7)]. This moderate decrease of TVOCs compared to higher‐emitting OSB was mainly due to lower concentration of terpenes (5100 μg/m^3^, day 1). In particular, whereas α‐pinene on day 1 was only slightly lower (3568 μg/m^3^), 3‐carene was significantly lower (485 μg/m^3^) compared to higher‐emitting OSB. Contrarily, the concentrations of specific terpenes on days 3 and 7 were similar in higher‐ and lower‐emitting OSB. As expected [7, 8], long‐term storage slightly increased the concentration of saturated aldehydes, especially hexanal, despite it occurring at low temperature. In fact, we recorded a slight (but significant on day 1) increase of hexanal [1086 μg/m^3^ (day 1), 1163 μg/m^3^ (day 3)] and of unsaturated aldehydes on days 3 and 7 [37 μg/m^3^ (day 3), 22 μg/m^3^ (day 7)] compared to higher‐emitting OSB. Lastly, organic acids, mainly represented by acetic (87%) and propionic (11%) acid, were relatively constant during exposure to both higher‐ and lower‐emitting OSB and present in similar amounts compared to cages without OSB (Figure S2A,B, respectively), pointing to a mouse excretion‐driven source.

Taken together, storage of OSB caused a substantial decrease in the concentration of terpenes, mainly of 3‐carene, and a barely significant increase of hexanal in cage air.

### 3.2 Higher‐Emitting OSB Enhances Some Inflammatory Parameters in ad

**FIGURE 2:**
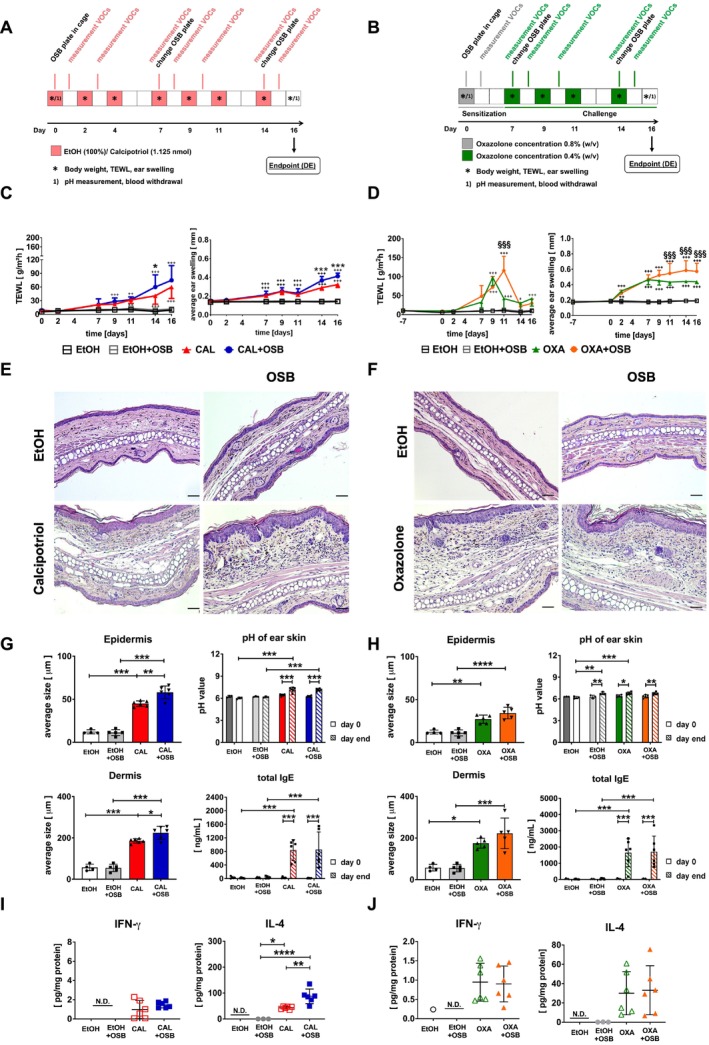
Influence of higher‐emitting OSB on two AD mouse models. Experimental set‐up for calcipotriol (A) or oxazolone‐induced (B) model on mouse ears and exposure to higher‐emitting OSB. TEWL (left), ear swelling (right) measurements for calcipotriol (C) or oxazolone (D) model. Histology (H&E) and size of skin layers of mouse ears treated with calcipotriol (E, G, left panels) or oxazolone (F, H, left panels). Scale bar: 50 μm. Skin pH (upper) and serum IgE (lower graphs) at day 0 and day end in calcipotriol‐ (G, right panels) or oxazolone‐treated mice (H, right panels). Cytokines in ear tissue lysates in the calcipotriol (I) or oxazolone (J) model. One representative experiment out of two, data are expressed as mean ± SD. C, D, *n* = 5‐6/group (*n* = 5: EtOH); G, H, *n* = 4–6/group (*n* = 4: EtOH); I, J, *n* = 6/group. One‐way ANOVA (G, H, left panels; I, J) and two‐way ANOVA (C, D; G, H, right panels) with Bonferroni post‐test. TEWL and ear swelling: ^++^ *p* < 0.01; ^+++^ *p* < 0.001 vs. EtOH; *p < 0.05 vs. calcipotriol; ^§§§^ *p* < 0.001 vs. oxazolone. Further graphs: *p < 0.05; **p < 0.01; ***p < 0.001; ****p < 0.0001. CAL, calcipotriol; OXA, oxazolone.

Two different ad models, a non‐antigen‐specific (calcipotriol, Figure 2A) and an antigen‐specific (oxazolone, Figure 2B), were employed. Transepidermal water loss (TEWL), widely used for assessing skin barrier function in humans and mice [28, 29] and ear swelling [28] were used to monitor disease progression. Application of both substances induced a typical increase in TEWL and ear thickness compared to control EtOH (Figure 2C,D) [28]. Interestingly, higher OSB emissions increased TEWL and ear thickness in the calcipotriol model from day 14 onwards, whereby the increase in TEWL was statistically significant only at day 14 (Figure 2C). In the oxazolone model, higher OSB emissions caused a retarded increase in TEWL, reaching the peak at day 11 instead of day 9 depicted for oxazolone, followed by a rapid decrease in both groups by day 14 (Figure 2D, left). In the same model, ear swelling was increased by OSB emissions starting from day 11 (Figure 2D, right). Contrarily, OSB emissions had no effect on TEWL or ear swelling in control EtOH animals in either model. Body weight was not affected in any treatment group (Figure S3A,B). Histological analysis of ear tissue from both models displayed typical hallmarks of ad, including parakeratosis, spongiosis, and inflammatory cell infiltration rich in macrophages (Figure S4), CD4^+^ lymphocytes, and eosinophils (Figure 2E,F and Figures S4 and S5, insets) [28, 30]. Measurements of epidermis and dermis thickness revealed not only the typical increase in both ad models versus EtOH [28] but also an additional increase due to exposure to OSB emissions, albeit significantly only in the calcipotriol model (Figure 2G,H, left panels). Again, these effects were restricted only to ad models, as control EtOH animals displayed no alterations due to OSB emissions. Similarly to human ad [31], skin pH increased in both ad models from an average of 6.4 to 7.2 and from 6.4 to 6.7 for calcipotriol and oxazolone, respectively (Figure 2G,H, right). High‐emitting OSB had no effects on skin pH in either disease model but led to an increase in pH from an average of 6.2 to 6.7 only in EtOH+OSB of the longer oxazolone model (Figure 2H, right). Also, serum IgE, notably increased in both ad models similarly to human ad [32], was not subjected to variations due to additional higher‐emitting OSB exposure (Figure 2G,H, right).

To further characterise the nature of the skin inflammatory response, different cytokines and chemokines were measured in ear tissue lysates. Treatment with both ad inducers led to increased levels of almost all measured cytokines and chemokines compared to EtOH, albeit significantly only for IL‐4, IL‐10, and IL‐6 in the calcipotriol model (Figure 2I,J and Figure S6A,B). Interestingly, higher‐emitting OSB significantly increased IL‐4 and slightly increased IL‐10 and IL‐1β release only in calcipotriol‐treated (Figure 2I and Figure S6A), but had no effect on these parameters in oxazolone‐treated mice (Figure 2J and Figure S6B).

Overall, these data demonstrate that higher OSB emissions lead to a moderate enhancement of some ad parameters in both murine ad models.

### 3.3 Higher‐Emitting OSB Triggers Selected Lipid Mediators in the Skin

**FIGURE 3:**
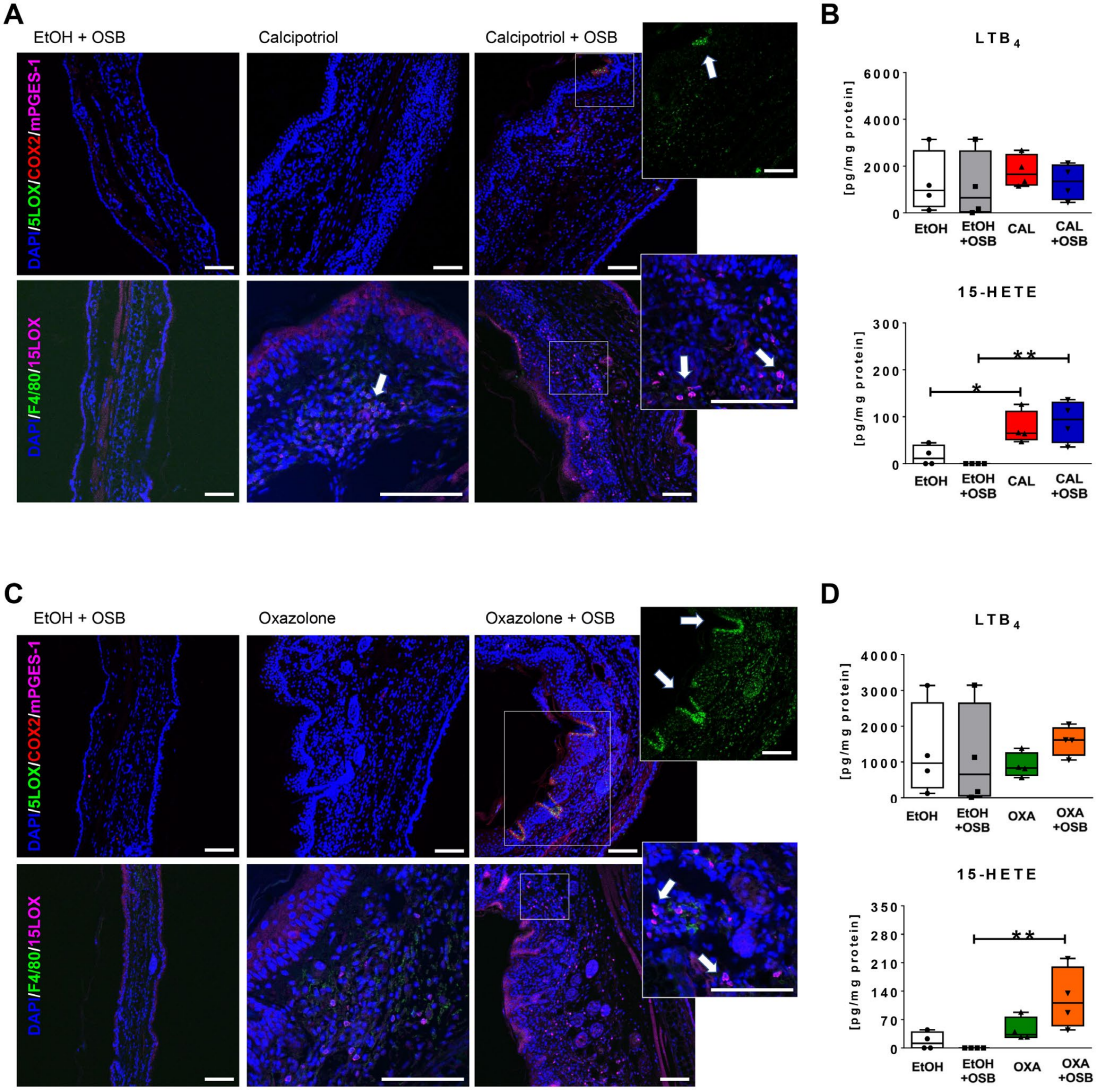
Higher‐emitting OSB drives pro‐inflammatory lipoxygenase pathways in AD. Immunofluorescent analysis of paraffin‐embedded sections of mouse ear treated with calcipotriol (A) or oxazolone (C) alone or with higher‐emitting OSB with relative EtOH + OSB control. Tissue sections were stained with antibodies against 5‐LOX, 15‐LOX, COX‐2, mPGES‐1, F4/80 and DAPI as indicated. Scale bar: 50 μm. Analysis of LTB_4_ and 15‐HETE in ear tissue lysates from calcipotriol (B) or oxazolone‐treated (D) animals, exposed or not to higher‐emitting OSB. Results expressed as boxplots indicating minimum, 25th percentile, median, 75th percentile, and maxi mum. *n* = 4/group. One‐way ANOVA with Bonferroni post‐test. *p < 0.05; **p < 0.01. CAL, calcipotriol; OXA, oxazolone.

Together with the inflammatory parameters described above, eicosanoids, particularly metabolites derived from the 5‐ and 15‐LOX pathways, as well as prostanoids (derived from the COX pathway), have been implicated in the pathophysiology of ad in humans [18, 33, 34, 35]. Immunofluorescent staining of COX‐2 and mPGES‐1, the enzymes generating PGE_2_, on ear skin of both ad models revealed COX‐2 and mPGES‐1‐positive cells in both ad models (Figure 3A,C, upper panels), independently from the presence of OSB emissions. On the contrary, whilst few 5‐LOX‐positive cells were detected in the calcipotriol model exposed to OSB (Figure 3A, upper panel, inset, arrow), large numbers of 5‐LOX‐positive cells, including epithelial cells and leukocytes, were detected following OSB exposure in the oxazolone model (Figure 3C, upper panel, inset, arrows). Furthermore, 15‐LOX‐positive cells, presumably (F4/80‐negative) eosinophils, present in both ad models, were increased following OSB exposure (Figure 3A,C, lower panels, insets, arrows). Quantification of LTB_4_ and 15‐HETE in skin homogenates revealed no variation for LTB_4_ between the four experimental groups (Figure 3B,D, top), whilst the level of 15‐HETE was increased in calcipotriol‐treated mice compared to respective EtOH controls (Figure 3B,D, bottom). OSB emissions showed a slight additive effect on 15‐HETE production in the calcipotriol model. In contrast, in the oxazolone model, 15‐HETE amounts in the skin were only significantly increased following exposure to OSB emissions, but not by oxazolone exposure alone. Thus, VOCs from OSB may elevate pro‐inflammatory lipid mediators, which contribute to tissue inflammation in ad.

### 3.4 Higher‐Emitting OSB Triggers Eosinophils in the Airways of Calcipotriol‐Treated Mice

**FIGURE 4:**
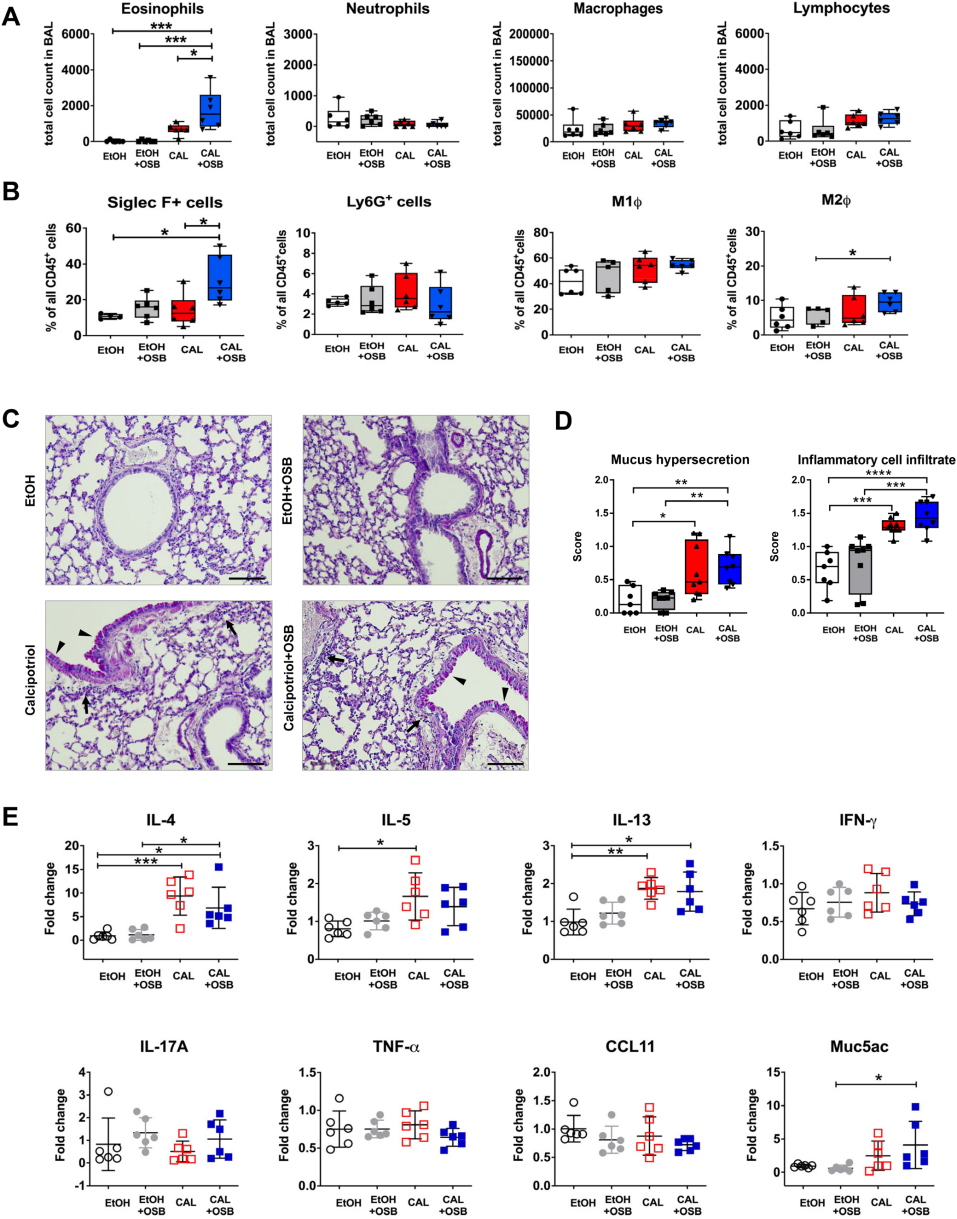
Influence of higher‐emitting OSB on lungs of calcipotriol‐treated animals. Lungs were retrieved from calcipotriol‐treated mice exposed or not to higher‐emitting OSB during the whole experiment. Analysis of BAL cellular infiltration (A), and flow cytometric analysis of lung tissue (B). Representative periodic acid–Schiff (PAS)‐stained lung sections (C). Arrows: Inflammatory infiltrate; arrowheads: Mucus hypersecretion; scale bar: 100 μm. Histological scoring (D). Expression of cytokines in lung tissue lysates evaluated by real‐time PCR (E). One representative experiment out of two, *n* = 6 mice/group. D, *n* = 7‐9/group. Results expressed as boxplots indicating minimum, 25th percentile, median, 75th percentile, and maximum or as mean ± SD. One‐way ANOVA with Bonferroni post‐test. *p < 0.05, **p < 0.01, ***p < 0.001, ****p < 0.0001. CAL, calcipotriol.

Having demonstrated that higher‐emitting OSB worsen skin barrier function in ad models, we sought to evaluate whether it would also impact asthma development following ad, a phenomenon known as atopic march [12, 36]. For this purpose, we analysed the lungs retrieved from calcipotriol/EtOH‐treated animals with/without OSB exposure. Interestingly, analysis of BAL inflammatory cell infiltration revealed a significant increase in eosinophils in calcipotriol‐treated animals exposed to higher‐emitting OSB compared to all other groups. On the contrary, infiltrations of neutrophils, macrophages, and lymphocytes remained unchanged (Figure 4A). On the same line, lung cellular infiltration evaluated by flow cytometry showed increased percentages of SiglecF^+^ cells in calcipotriol+OSB lungs compared to calcipotriol or EtOH. Similarly to the BAL, no differences in the percentages of Ly6G^+^, CD4^+^, CD8^+^ T cells, and M1 macrophages were detected in the lungs, only enhanced M2 macrophages in calcipotriol‐treated mice exposed to higher‐emitting OSB compared to respective EtOH controls (Figure 4B and Figure S7). Lung histological analysis showed a slight but significant increase in mucus hypersecretion and inflammatory cell infiltrate in calcipotriol‐treated animals compared to EtOH, whereby OSB emissions showed no effect (Figure 4C,D). To further characterise the type of lung inflammatory response, cytokine expression was analysed in lung tissue. Treatment with calcipotriol increased lung expression of the Th2 cytokines *IL4*, *IL5*, and *IL13*, whereas no difference was detected for *IFN*γ, confirming a type‐2 immunological response. OSB exposure had no effect on Th2 cytokine expression. Other lung inflammatory cytokines showed no regulation by calcipotriol or OSB, only *Muc5ac* was upregulated in the lungs of calcipotriol+OSB animals versus respective EtOH controls (Figure 4E). Overall, we show that treatment with calcipotriol on ear skin evokes a type‐2 immunologic response in mouse lungs and that exposure to higher‐emitting OSB increases lung eosinophil recruitment.

### 3.5 Lower‐Emitting OSB has Even Beneficial Effects in Oxazolone‐Treated Mice

**FIGURE 5:**
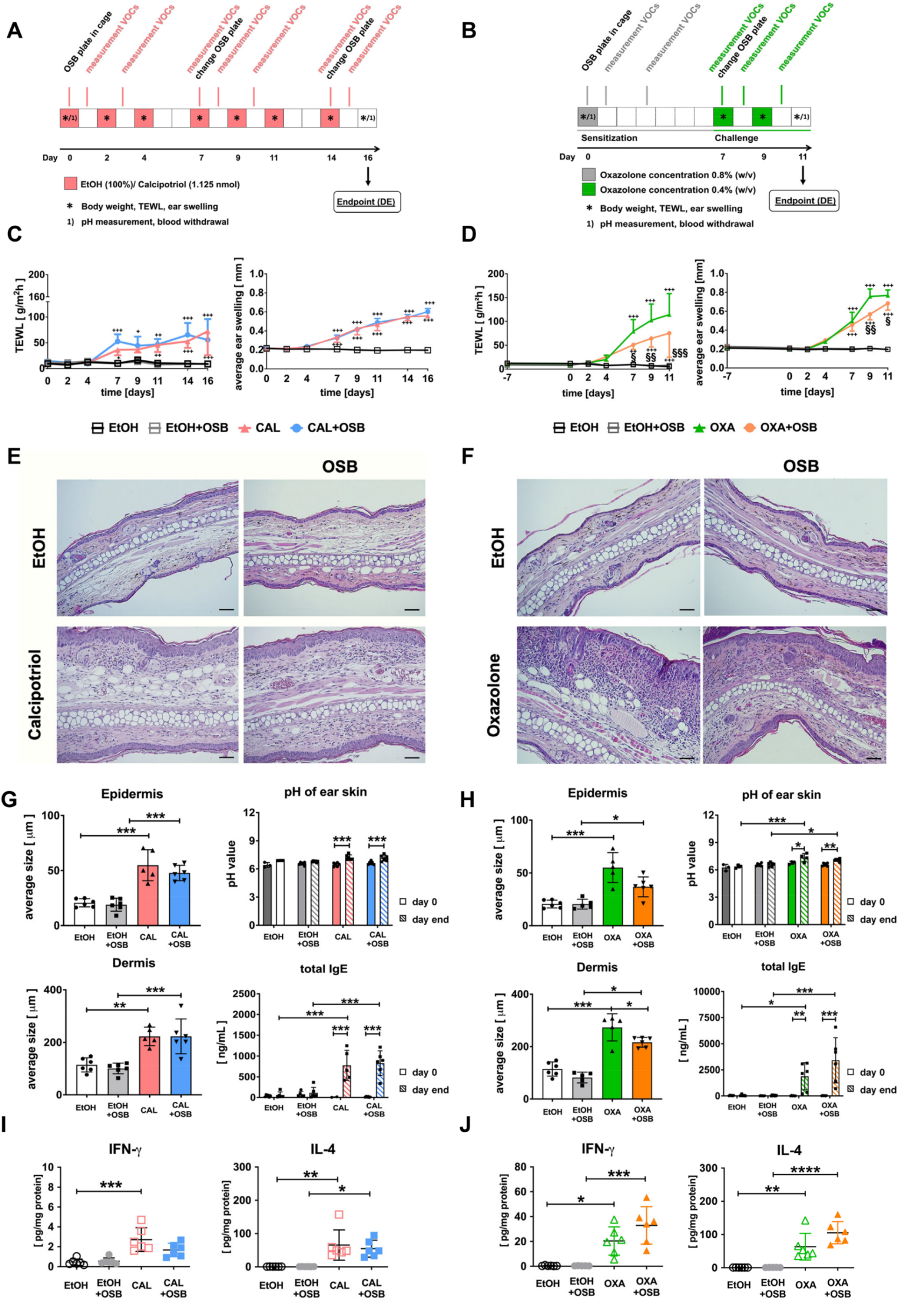
Influence of lower‐emitting OSB on two AD mouse models. Experimental set‐up for calcipotriol (A) or oxazolone‐induced (B) model on mouse ears and exposure to lower‐emitting OSB. TEWL (left) and ear swelling (right) measurements for calcipotriol (C) or oxazolone (D) model. Histology (H&E) and size of epidermis and dermis of mouse ears treated with calcipotriol (E, G, left panels) or oxazolone (F, H, left panels). Scale bar: 50 μm. Skin pH (upper graphs) and serum IgE (lower graphs) at day 0 and day end in calcipotriol‐ (G, right panels) or oxazolone‐treated mice (H, right panels). Cytokines measured in ear tissue lysates in the calcipotriol (I) or oxazolone (J) model. One representative experiment out of two. Data are expressed as mean ± SD, *n* = 5–6/group. One‐way ANOVA (G, H, left panels; I, J) and two‐way ANOVA (C, D; G, H, right panels) with Bonferroni post‐test. TEWL and ear swelling: ^+^ *p* < 0.05; ^++^ *p* < 0.01; ^+++^ *p* < 0.001 vs. EtOH; ^§^ *p* < 0.05; ^§§^ *p* < 0.01; ^§§§^ *p* < 0.001 vs. oxazolone. Further graphs: *p < 0.05; **p < 0.01; ***p < 0.001; ****p < 0.0001. CAL, calcipotriol; OXA, oxazolone.

**FIGURE 6:**
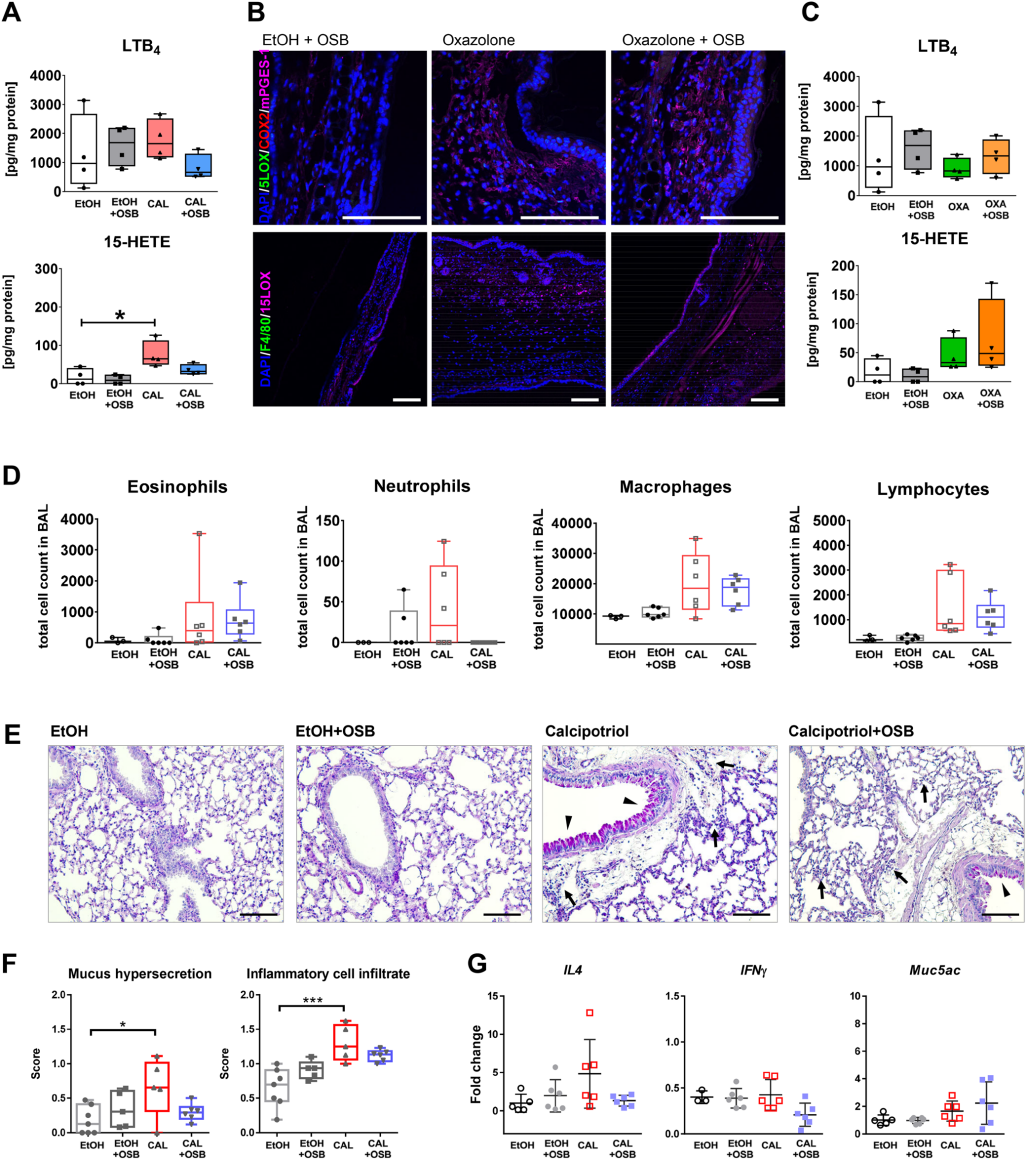
Effect of lower‐emitting OSB on skin lipid mediators and lung inflammatory response in AD models. LTB_4_ and 15‐HETE were measured in ear lysates of calcipotriol (A) or oxazolone‐treated (C) animals and respective EtOH controls with/without lower‐emitting OSB. Immunofluorescent analysis of mouse ear sections treated with oxazolone with/without lower‐emitting OSB (B). Stainings for 5‐LOX, 15‐LOX, COX‐2, mPGES‐1, F4/80 and DAPI, as indicated. Scale bar: 50 μm. BAL cellular infiltration from lungs of calcipotriol‐treated mice with/without lower‐emitting OSB (D) and representative periodic acid–Schiff (PAS)‐stained lung sections (E). Arrows: Inflammatory infiltrate; arrowheads: Mucus hypersecretion; scale bar: 100 μm. Histological scoring (F). Cytokine expression in lung tissue lysates evaluated by real‐time PCR (G). One representative experiment out of two. A, C: *N* = 4; D–G: *N* = 3–7 mice/group. Results expressed as boxplots indicating minimum, 25th percentile, median, 75th percentile, and maximum or as mean ± SD. One‐way ANOVA with Bonferroni post‐test. *p < 0.05; ***p < 0.001. CAL, calcipotriol; OXA, oxazolone.

Contrarily to the effects shown for higher‐emitting OSB (Figure 3), exposure to lower‐emitting OSB had no effect on TEWL or ear thickness in the calcipotriol model and even significantly reduced both parameters in oxazolone‐treated animals (Figure 5C,D). Mouse body weight was again unaffected in both models (Figure S3C,D). Histological analysis of ear tissue showed that exposure to lower‐emitting OSB had no effects in the calcipotriol model [Figure 5E,G (left panels)], whereas beneficial effects in terms of epidermal and dermal thickness and inflammatory cell infiltration were detected in the oxazolone model [Figure 5F,H (left panels)]. As for higher‐emitting OSB, skin pH and serum IgE were not altered by lower‐emitting OSB (Figure 5G,H, right panels). Furthermore, lower‐emitting OSB had no effect on the levels of either cytokines, chemokines (Figure 5I,J and Figure S6C,D) or LTB_4_ and 15‐HETE (Figure 6A,C) measured in skin homogenates of both models. Notably, exposure to lower‐emitting OSB in oxazolone‐treated animals failed to induce increased 5‐LOX and 15‐LOX expression (Figure 6B), in contrast to the results shown for higher‐emitting OSB (Figure 3). On the same line and contrarily to the effects shown for higher‐emitting OSB (Figure 4), analysis of BAL retrieved from lungs of calcipotriol‐treated animals exposed to lower‐emitting OSB showed no additional effect due to OSB (Figure 6D). Similarly to higher‐emitting OSB (Figure 4), lower‐emitting OSB had no effect on lung histology or cytokine expression (Figure 6E–G). Therefore, exposure to lower‐emitting OSB had no effect on the calcipotriol‐induced skin and lung phenotype and had even beneficial effects on oxazolone‐treated animals.

To translate our findings to humans, we employed an in vitro model of human keratinocytes incubated with T cell supernatant (TCS) generated from lesional skin of atopic dermatitis patients and exposed them to a specific VOC mix simulating lower‐emitting OSB (Table S3 and Figure S8A). TCS alone induced a significant increase of IL‐1β and a slight increase of CCL‐2 compared to control. Interestingly, OSB emissions significantly reduced the release of CCL2 from TCS‐stimulated and unstimulated keratinocytes and slightly reduced IL‐1β from TCS‐stimulated keratinocytes (Figure S8B), confirming no or rather beneficial effects of OSB‐specific VOCs in a human in vitro atopic dermatitis model.

## 4 Discussion

Wood and wood‐based materials, because of their characteristics of being renewable, recyclable, and reusable, are sustainable products vastly used in construction and furniture. The resulting VOC emissions, accumulating in indoor air, can potentially become dangerous for human health [2, 37, 38]. Data from the German Federal Environmental Agency, based on a survey of 479 households, defined indoor TVOC levels below 1 mg/m^3^ as good air quality, between 3 and 10 mg/m^3^ as critical, and above 10 mg/m^3^ as unacceptable [39], without including health risk assessments. Although indoor TVOC values in residential homes typically fall within the good air quality range [40], interestingly, in new or newly renovated buildings, including timber houses, TVOC concentrations can exceed 3 mg/m^3^, thus falling in the critical air quality range [9, 41]. Whereas OSB emissions above 10 mg/m^3^, measured here only for single higher‐emitting OSB boards, can be realistic only in particular occupational settings (e.g. in production facilities) [42], the level of TVOCs recorded for lower‐emitting OSB in mouse cage models concentrations measurable either in occupational settings or in newly built timber houses [9, 42]. By exchanging the OSB plates once a week, we maintained a critical TVOC concentration range in mouse cages during most of the duration of the experiments. To take into consideration the versatility of ad [15] and to disentangle the effects of VOC exposure, we used two different, rather mild murine models of the disease [28] and combined them with OSB emissions under controlled exposure conditions. Placing the OSB at the bottom of the cage and avoiding their direct contact with the animals, we circumvented additional substance applications to the treated skin as performed in analogous studies [43].

Our results show that exposure to higher‐emitting OSB during the development of ad affected skin barrier function, as it was shown in human exposure studies employing different VOCs or VOC mixtures [27, 44]. Additionally, higher‐emitting OSB induced ear swelling and local release of a few distinct cytokines (i.e., IL‐4, IL‐1β) only in the calcipotriol model. Contrarily, we did not detect OSB‐induced effects on skin pH or serum IgE. In line with the eosinophilia detected in the skin in both models by histological analysis, we revealed an increased number of F4/80^−^, 15‐LOX^+^ cells, most likely representing eosinophils, conforming to the abundant 15‐LOX^+^ eosinophils accumulating in type 2 immune contexts [45, 46]. Indeed, higher concentrations of OSB emissions increased 15‐HETE production in ad skin, correlating with the increased accumulation of eosinophils triggered by exposure to higher‐emitting OSB. Whether 15‐LOX‐expressing eosinophils in the skin are major drivers of epidermal thickening and ad development in our models should be a matter of future investigations, especially considering the important role of eosinophils in atopy risk assessment in the context of VOC exposure [47]. Contrarily to 15‐LOX, the expression of 5‐LOX differed in the two models. While the calcipotriol model accumulated very few 5‐LOX^+^ cells with no variations in LTB_4_ production, 5‐LOX^+^ cells accumulated in the oxazolone model following higher‐emitting OSB exposure. In contrast to a previous study [48], we were unable to detect increased LTB_4_ concentrations in the oxazolone model, possibly due to highly variable LTB_4_ baseline levels in non‐inflamed skin in our hands.

To investigate if higher‐emitting OSB impacts the atopic march, we chose the calcipotriol model as this model was proven to aggravate ovalbumin‐induced airway inflammation in mice [49]. Strikingly, exposure to higher concentrations of OSB emissions without co‐exposure to a respiratory allergen led to an increased infiltration of eosinophils in the lungs of mice subjected to the ad model, suggesting that exposure to OSB emissions may drive the atopic march. Other than that, no further increase in lung inflammatory parameters was detected, probably due to the relatively early time point of sacrifice.

Surprisingly, the employment of lower‐emitting OSB, characterised by reduced TVOC concentrations, especially of 3‐carene after long‐term storage, abolished the pro‐inflammatory effects observed using higher‐emitting OSB in both models, even converting its effects to beneficial in the oxazolone model. Additionally, lower‐emitting OSB failed to induce the BAL eosinophilia observed with higher‐emitting OSB in calcipotriol‐treated animals. These results fit into the controversial discussion about the influence of individual VOCs analysed in different disease models, acting either as aggravating for the disease [27] or rather as inert or even anti‐inflammatory [20, 50, 51, 52]. In particular, data on health effects of specific VOC compounds such as 3‐carene is scanty. This terpene is vastly found as an ingredient in cosmetics, paints, and varnishes or in various household products [53]. Whilst 3‐carene contained in cosmetic products seems to lack skin sensitisation properties [54], its oxidised form seems to have an impact in turpentine allergy [55] and could therefore (alone or in conjunction with other OSB‐specific VOCs) play a role in our model. Nevertheless, it is important to note that the translation of our in vivo data to humans necessitates careful consideration due not only to physiological and anatomical differences between mice and humans, but also to the distinct metabolism of single VOCs. Therefore, to attempt a translation to humans, we exposed an in vitro model for humans to OSB‐specific emissions. Although we show only a slight reduction in IL–1β release, OSB‐specific VOCs induced a significant decrease of CCL2, a key chemokine involved in monocyte/macrophage infiltration in inflammatory skin diseases [56, 57], a hint towards potential beneficial effects of OSB emissions in humans.

## 5 Conclusion

Taken together, this study demonstrates that higher‐emitting OSB, characterised especially by elevated concentrations of 3‐carene, may worsen the development of ad and increase lung eosinophil recruitment. Contrarily, lower concentrations have no or even beneficial effects on disease development. Assessing relative concentrations of individual VOCs and identifying the respective emitting sources could eventually be important for policymakers to define the prolonged storage time of each source, thus achieving improved disease control and prevention, particularly in early life.

## Author Contributions

The mouse experimental work was conceptualised by Stefan Haak and Francesca Alessandrini, and the human in vitro study by Stefanie Eyerich and Francesca Alessandrini. The experimental investigations were carried out by Katja Butter (VOCs measurements); Evelyn Schneider, Benjamin Schnautz, Stephanie Musiol, Johanna Grosch, Francesca Alessandrini (in vivo experiments); Stephanie Musiol and Manuel Garcia‐Käufer (in vitro experiments); Sonja Schindela and Julia Esser‐von Bieren (immunofluorescence). Data visualisation was carried out by Evelyn Schneider, Katja Butter, Julia Esser‐von Bieren, and Francesca Alessandrini, and analysis by Evelyn Schneider, Katja Butter, Francesca Alessandrini. The experimental investigations were supervised by Richard Gminski, Martin Ohlmeyer, Carsten B. Schmidt‐Weber, Stefanie Eyerich, Julia Esser‐von Bieren, and Francesca Alessandrini. Funding acquisition for this study was applied by Richard Gminski, Stefan Haak, Martin Ohlmeyer, Carsten B. Schmidt‐Weber, Stefanie Eyerich, and Francesca Alessandrini, and responsible for project administration was Francesca Alessandrini. The manuscript was written by Evelyn Schneider, Katja Butter, Manuel Garcia‐Käufer, Stefanie Eyerich, and Francesca Alessandrini, and was read, edited, and approved by all authors.

## Ethics Statement

All murine experiments were carried out under federal guidelines for the use and care of laboratory animals and approved by the government of the district of upper Bavaria (Approval n. ROB‐55.2‐2532.VET_02‐16‐198). For the human in vitro study, isolation of primary keratinocytes was approved by the local ethical committee of the Klinikum rechts der Isar, Technical University of Munich, Project number 5590/12 and 44/16 S.

## Conflicts of Interest

The authors declare no conflicts of interest.

## Supporting information


Figure S1–S8.

Table S1–S3.

Data S1.

## Data Availability

The data generated and analysed during this study are included in this published article and in its additional files.
