# Supplementary material for: Effects of Emissions From Oriented Strand Board on the Development of Atopic Dermatitis Using Two Different Experimental Mouse Models
Source: Exp Dermatol. 2025 Mar 20;34(3):e70086. doi: 10.1111/exd.70086 (PMC11926298; doi:10.1111/exd.70086)
Supplement: Supplementary file 1 — Figure S1–S8. [file EXD-34-e70086-s003.pdf]

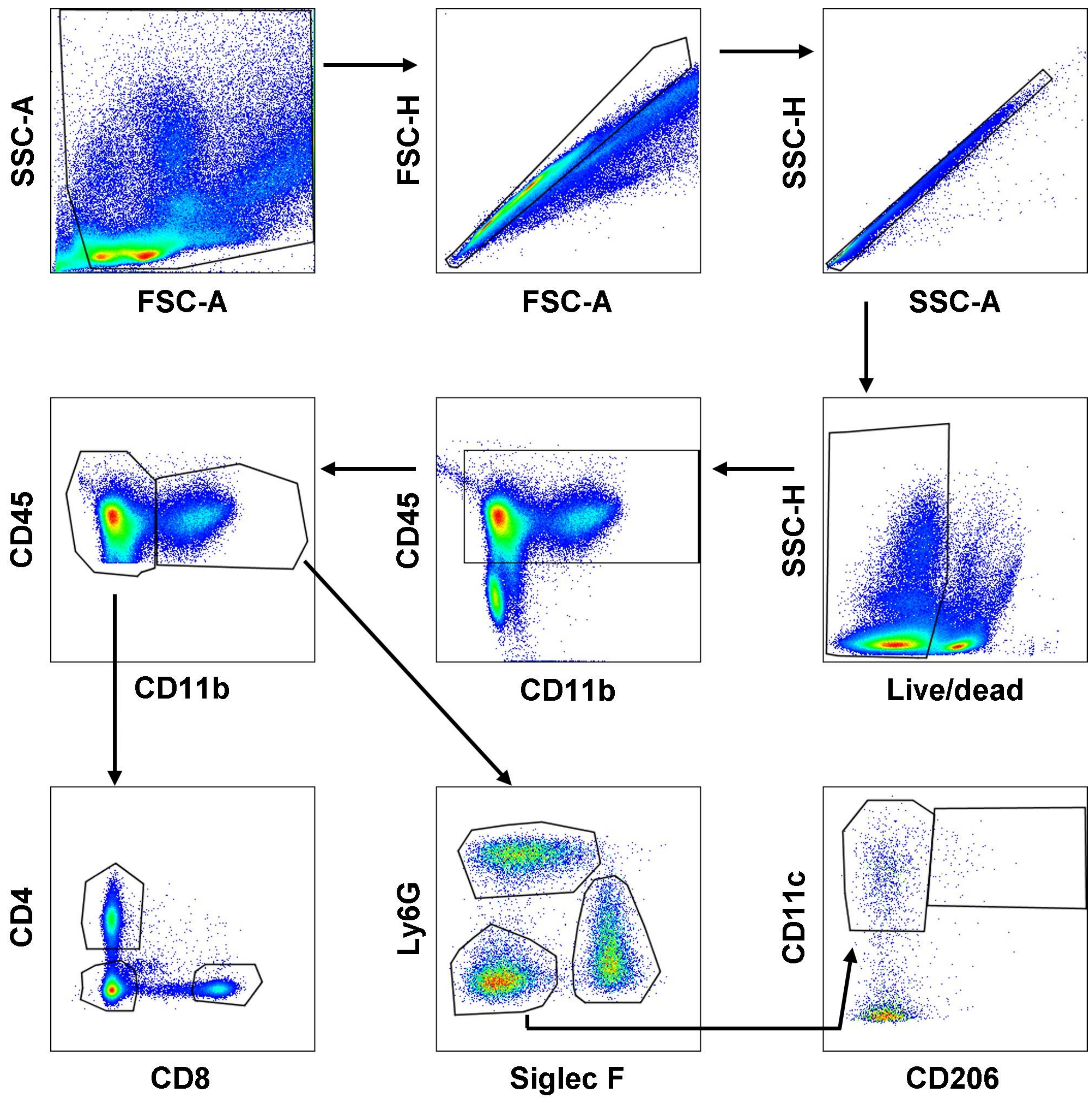

Figure S1. Gating strategy for the identification of specific cell populations in mouse lungs.

Gating strategy is shown for $CD4^{+}$ or $CD8^{+}$ T cells, $SiglecF^{+}$ eosinophils, $Ly6G^{+}$ neutrophils and $CD11c^{+}$ $CD206^{+}$ macrophages isolated from lung tissue samples.

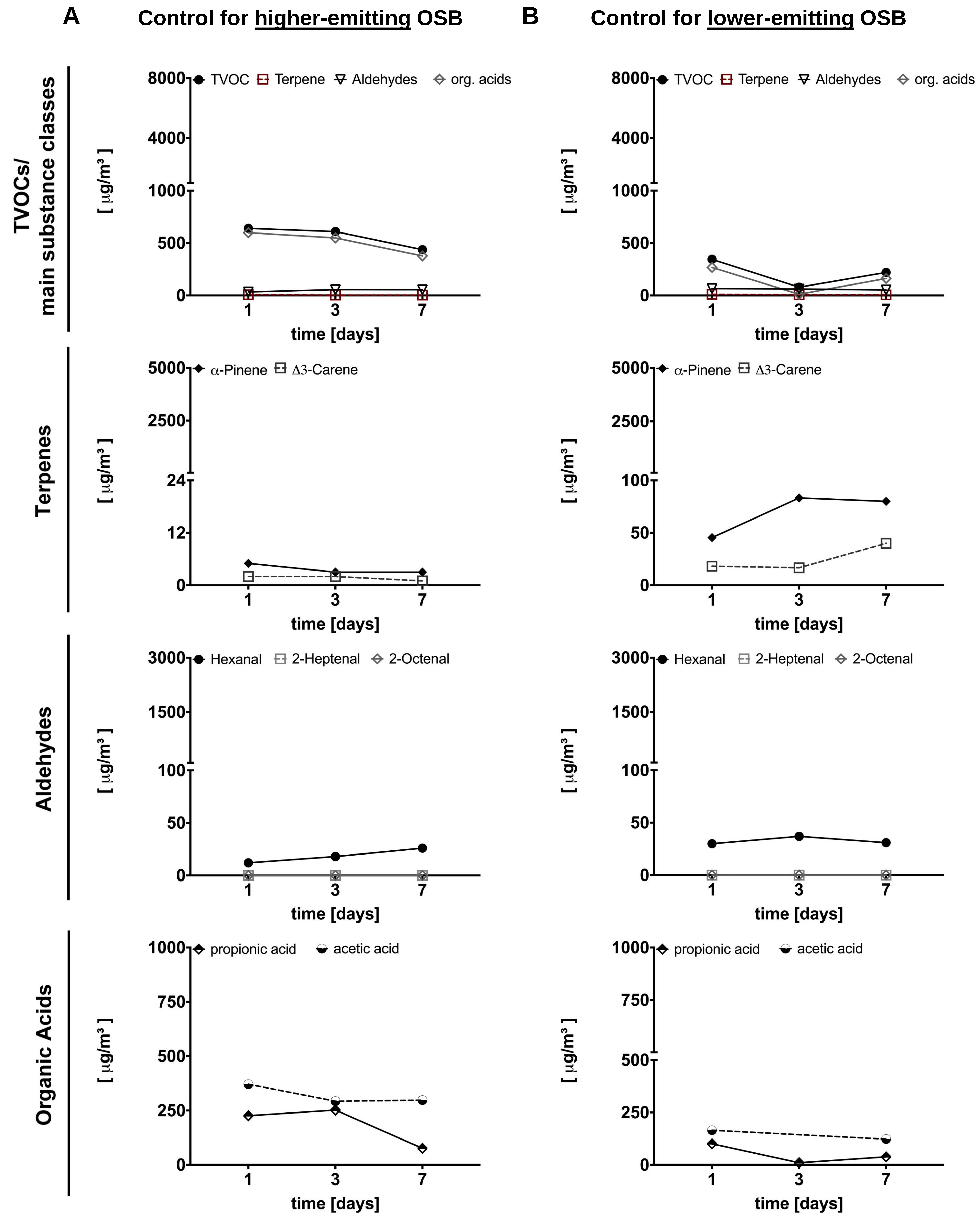

Figure S2. VOC emissions measured in control cages (without OSB)

VOC monitoring was carried out 3 times a week. The graphs depict cumulative TVOC concentrations, and subsequent main representatives for terpenes, aldehydes and organic acids measured in cages without OSB as controls for experiments with higher-emitting **(A)** or lower-emitting **(B)** OSB. Data represent concentrations of compounds measured during one week of exposure for one representative experiment.

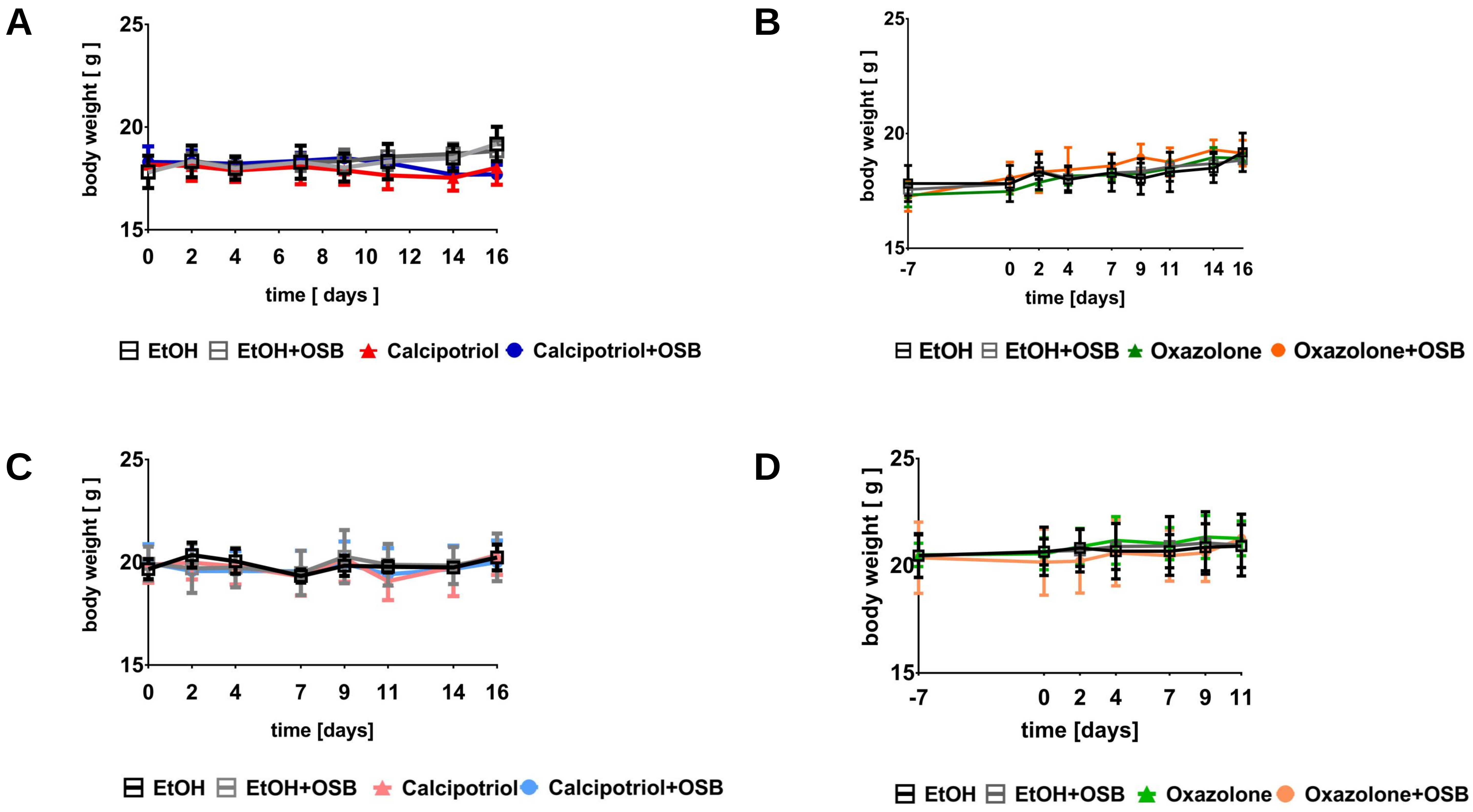

Figure S3. Measurements of mouse body weight

C57BL/6J mice were treated either with calcipotriol **(A, C)**, oxazolone **(B, D)** or EtOH control and exposed to higher OSB emissions **(A, B)** or to lower OSB emissions **(C, D)**. Body weight was monitored throughout all experiments. Each data point represents the mean ± SD; n=5-6/group. Data display one representative experiment out of two.

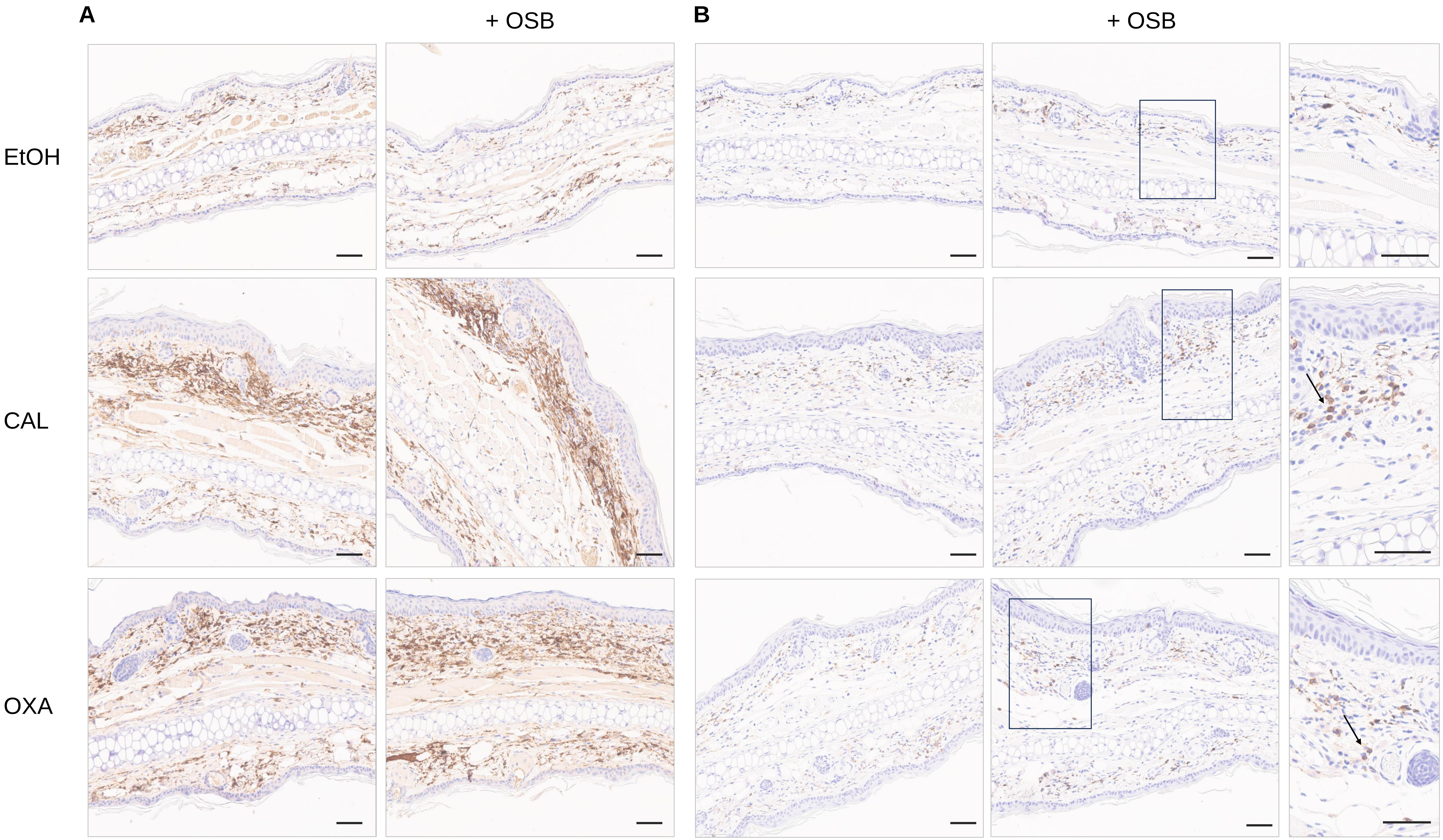

Figure S4. Immunohistochemical analysis of murine ear tissue

C57BL/6 mice were treated either with calcipotriol (CAL), oxazolone (OXA) or vehicle control (EtOH) and exposed (A, B, right panels) or not (A, B, left panels) to higher-emitting OSB. Immunohistochemical staining for macrophages (IBA-1), (A) and CD-4$^+$ T-helper cells (B). Scale bar: 60 µm.

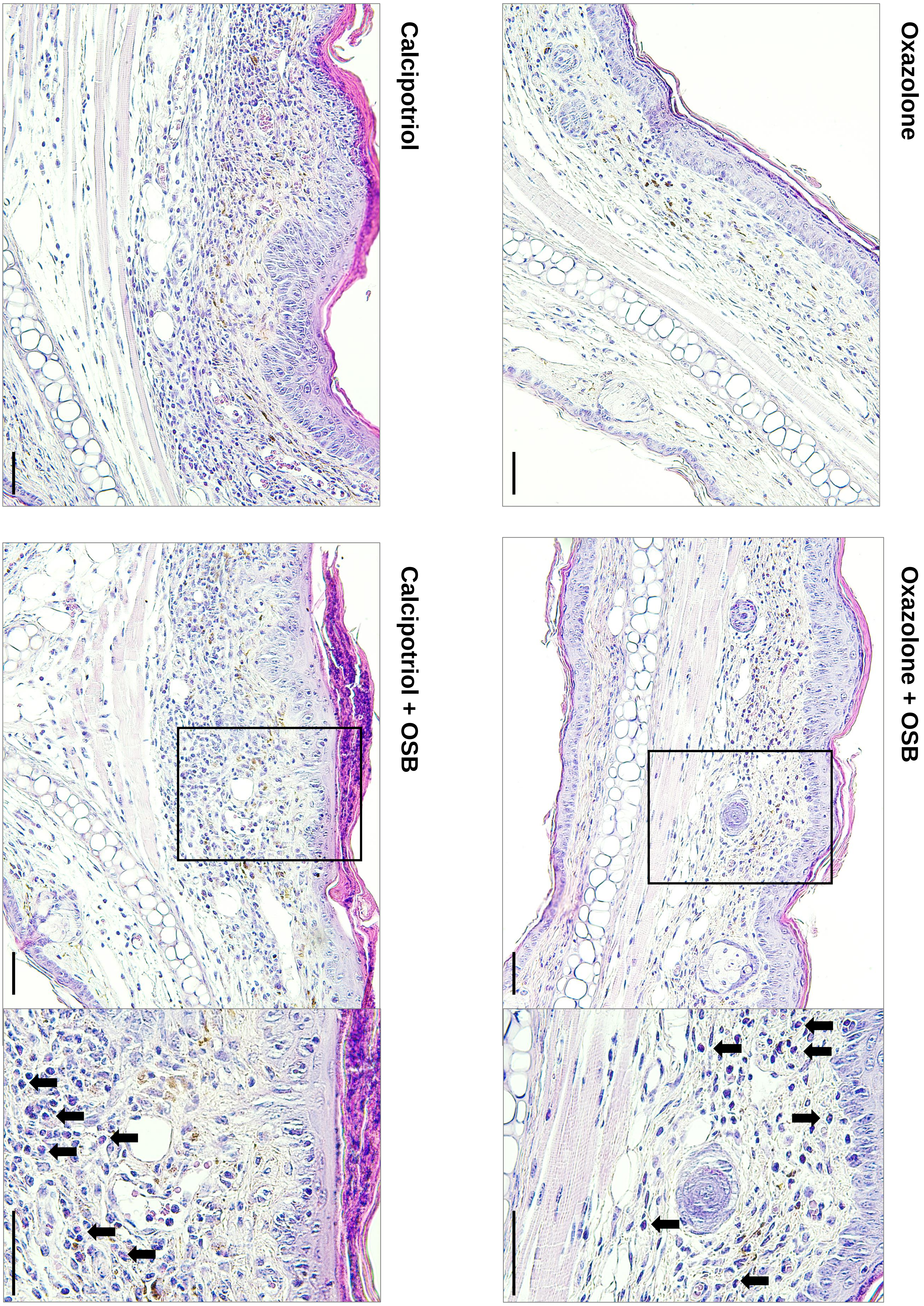

Figure S5. Histological analysis of murine ear tissue

C57BL/6J mice were treated either with calcipotriol (left) or oxazolone (right) and exposed to higher-emitting OSB. Histological localization of eosinophils on H&E stained-tissue sections (arrows). Scale bar: 50 µm.

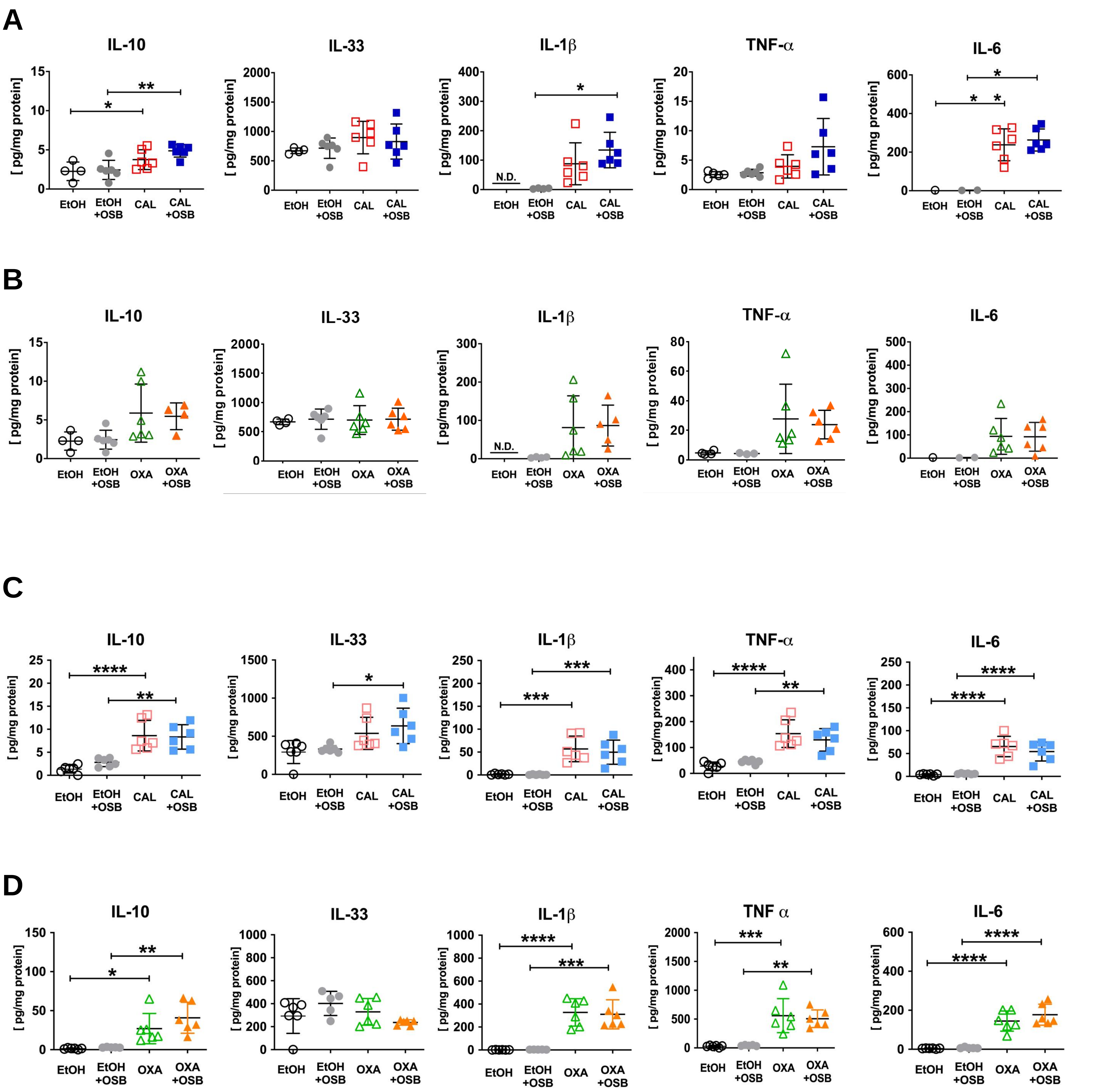

Figure S6. Pro-inflammatory cytokines expression in mouse ear homogenates

Cytokine expression was analyzed in ear tissue lysates of calcipotriol-treated **(A, C)** or oxazolone-treated **(B, D)** animals exposed to higher **(A, B)** or lower **(C, D)** OSB emissions. Each data point represents an individual mouse. A, B: n=4-6/group (n=4, EtOH). C, D: n=6/group. Data are expressed as mean ± SD. *P < 0.05; **P < 0.01; ***P < 0.001; ****P < 0.0001 (one-way Anova with Bonferroni posttest). CAL, calcipotriol; OXA, oxazolone.

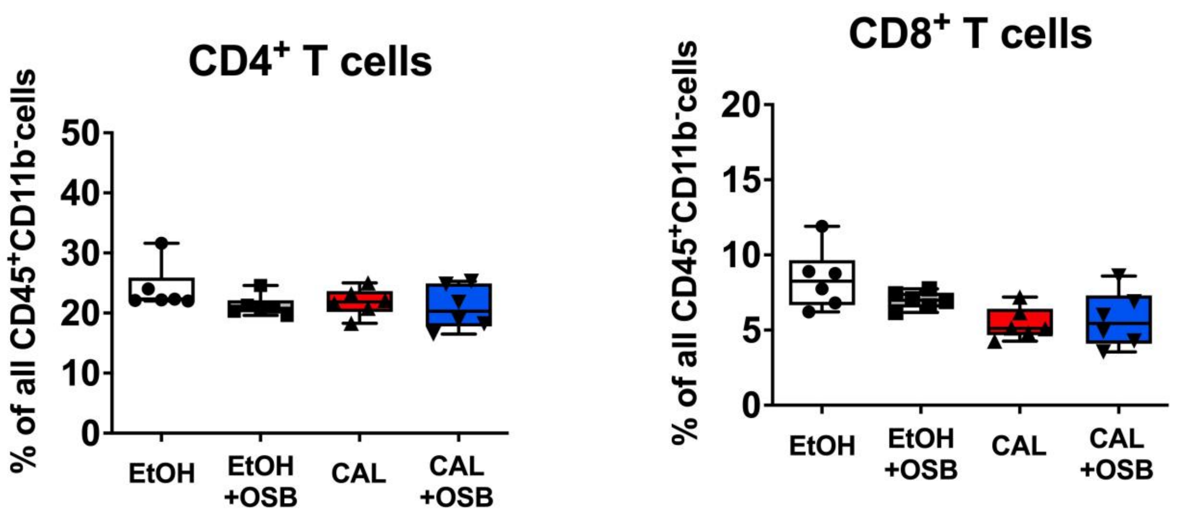

Figure S7. FACS analysis of lung tissue

Percentage of $CD4^+$ and $CD8^+$ T cells in lungs of calcipotriol-treated mice exposed to higher-emitting OSB evaluated by flow cytometry. Data display one representative experiment out of two. n=6/group. CAL, calcipotriol.

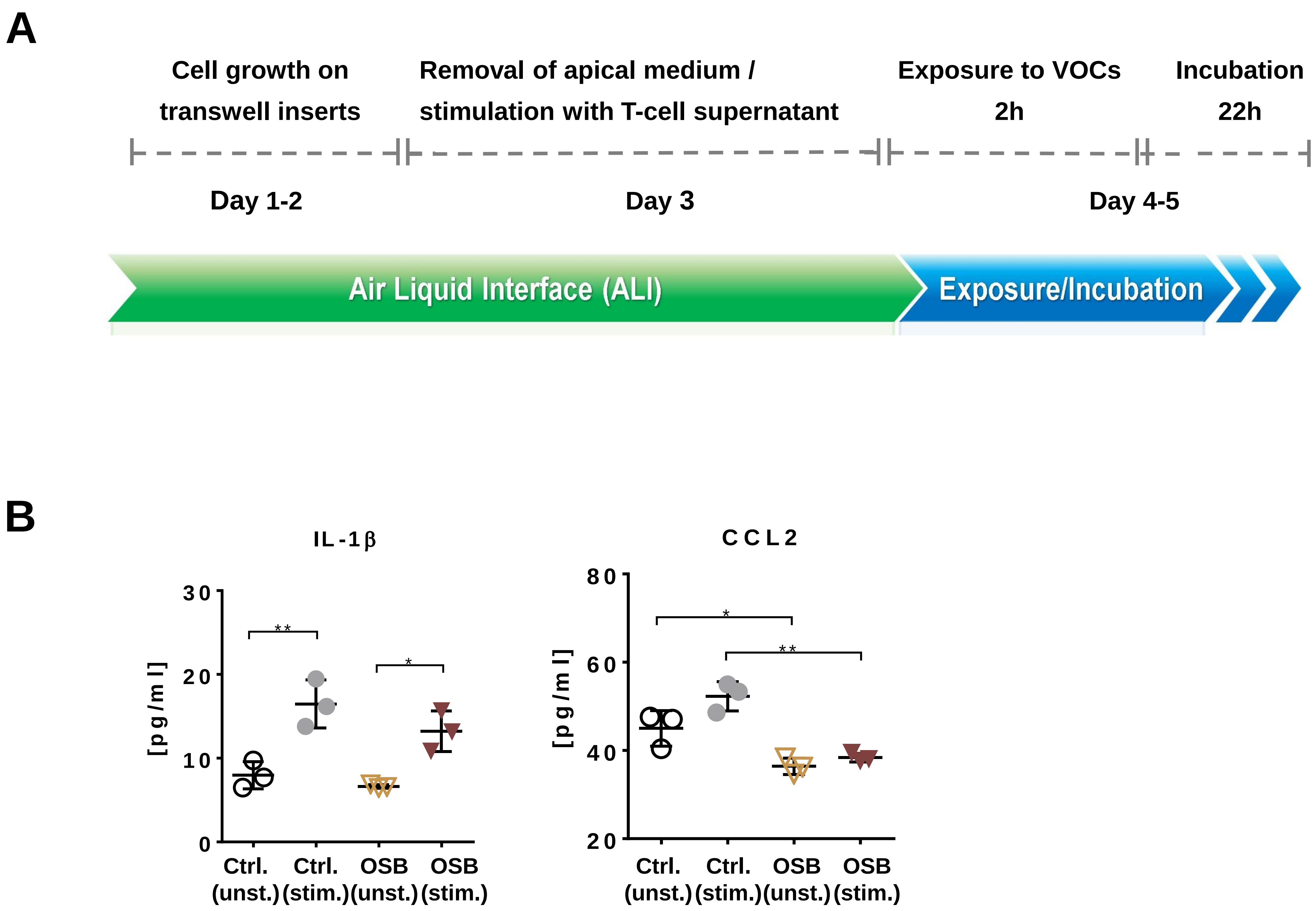

Figure S8. Influence of VOCs typical for OSB emission on a human *in vitro* model of AD

Human primary keratinocytes were cultivated at the air liquid (ALI) interface, stimulated for 12 h with T-cell supernatant, exposed for 2 h to a mixture of VOCs representative for OSB emission and post-incubated for additional 22 h at ALI conditions. Experimental scheme **(A)**. Cytokine analysis in cell culture supernatant at the end of experiment **(B)**. Each data point represents the mean of two replicates of exposures each of them analyzed in duplicates. n=3/group. Data are expressed as mean ± SD, one-way ANOVA with Bonferroni post-test. *P < 0.05, **P < 0.01. Ctrl, control exposed to clean air; OSB, exposed to synthetic OSB mixture; unst, non stimulated; stim, stimulated with T-cell supernatant
