# Supplementary material for: Effects of Emissions From Oriented Strand Board on the Development of Atopic Dermatitis Using Two Different Experimental Mouse Models
Source: Exp Dermatol. 2025 Mar 20;34(3):e70086. doi: 10.1111/exd.70086 (PMC11926298; doi:10.1111/exd.70086)
Supplement: Supplementary file 2 — Table S1–S3. [file EXD-34-e70086-s001.docx]

**Supplementary Tables**

**Table S1.** Antibodies used for flow cytometry of mouse lungs

| **Antigen** | **Fluorophor** | **Company** | **Dilution** |
| --- | --- | --- | --- |
| CD206 | PE-Dazzle594 | BioLegend | 1:500 |
| F4/80 | APC-e780 |  | 1:200 |
| Ly-6G | PB | BioLegend | 1:200 |
| Siglec-F | PE | BD | 1:300 |
| CD11b | BV711 | BioLegend | 1:700 |
| CD11c | PE-Cy7 | BioLegend | 1:300 |
| CD45 | PerCP-Cy5.5 | BD | 1:500 |
| CD4 | AF700 | BioLegend | 1:500 |
| CD8 | FITC | eBioscience | 1:600 |
| live/dead | AmCyan |  | 1:800 |

**Table S2.** Murine primers used for qPCR

| **Gene** | **Primer sequence** |
| --- | --- |
| *IL4* | 5'-GTCATCCTGCTCTTCTTTCTCG-3'  5'-CTCTCTGTGGTGTTCTTCGTTG-3' |
| *IL5* | 5'-AGCACAGTGGTGAAAGAGACCTT-3'  5'-TCCAATGCATAGCTGGTGATTT-3' |
| *IL13* | 5'-CCTGGCTCTTGCTTGCCTT-3'  5'-GGTCTTGTGTGATGTTGCTCA-3' |
| *IFNγ* | 5'-GCATTCATGAGTATTGCCAAG-3'  5'-GGTGGACCACTCGGATGA-3' |
| *IL17A* | 5'-ATCAGGACGCGCAAACATGA-3'  5'-TTGGACACGCTGAGCTTTGA-3' |
| *Tnfα* | 5'-TCTGTCTACTGAACTTCGGGGTGA-3'  5'-TTGTCTTTGAGATCCATGCCGTT-3' |
| *Ccl11* | 5'-CATCTGTCTCCCTCCACCAT-3'  5'-TAAAGCAGCAGGAAGAAGTTGGG-3' |
| *Muc5ac* | 5'-TGGAGTCAGCACGAAAACAG-3'  5'-GCACTGGGAAGTCAGTGTCA-3' |
| *GAPDH* | 5'- CGTCCCGTAGACAAAATGGT-3'  5'- TTGATGGCAACAATCTCCAC-3' |
| *β-actin* | 5'-TTCTTTGCAGCTCCTTCGTT-3'  5'-ATGGAGGGGAATACAGCCC-3' |

**Table S3**: VOC concentrations of reference compounds emitted from native lower-emitting OSB compared to concentrations of VOCs in exposure atmosphere used for the *in vitro* experiments

| **Reference compounds** | **CAS number** | **Concentration**  **μg/m^3^*** | **%*** | **Mass concentrations of reference compounds in gas-sampling bags (µg/L)** | **Conc. of VOCs in exposure atmosphere µg/L  (% recovery)**** |
| --- | --- | --- | --- | --- | --- |
| α-pinene | 80-56-8 | 1,649 | 55.37 | 2,769 | 978 / (35) |
| 3-carene | 13466-78-9 | 462 | 15.51 | 776 | 152 / (20) |
| limonene | 5989-27-5 | 23 | 0.77 | 39 | 5 / (13) |
| hexanal | 66-25-1 | 801 | 26.90 | 1,344 | 417 / (31) |
| 2-octenal | 2548-87-0 | 43 | 1.44 | 72 | 2 / (3) |
| Total |  | 2,978 | 100 | 5,000 | 1,554 / (31) |

* Data based on chemical analyses (TD-GC-MS) of representative VOCs emitted by native OSB

** VOC concentrations in exposure atmosphere were calculated by recovery experiments using GC-FID analyses. Percent recovery values for each VOC are given in brackets.
