# Supplementary material for: Effects of Emissions From Oriented Strand Board on the Development of Atopic Dermatitis Using Two Different Experimental Mouse Models
Source: Exp Dermatol. 2025 Mar 20;34(3):e70086. doi: 10.1111/exd.70086 (PMC11926298; doi:10.1111/exd.70086)
Supplement: Supplementary file 3 — Data S1. [file EXD-34-e70086-s002.docx]

**Supporting information**

**Effects of emissions from oriented strand board on the development of atopic dermatitis using two different experimental mouse models**

Evelyn Schneider^1*^, Katja Butter^2*^, Benjamin Schnautz^1^, Stephanie Musiol^1^, Johanna Grosch^1^, Sonja Schindela^1^, Manuel Garcia-Käufer^3^, Richard Gminski^3^, Stefan Haak^1^, Martin Ohlmeyer^2^, Carsten B. Schmidt-Weber^1,4^, Stefanie Eyerich^1^, Julia Esser-von Bieren^1^, Francesca Alessandrini^1^

^1^ Center of Allergy & Environment (ZAUM), Technical University of Munich (TUM) and Helmholtz Zentrum München, German Research Center for Environmental Health, Neuherberg, Germany

^2^ Thünen Institute of Wood Research, Hamburg, Germany

^3^ Institute for Infection Prevention and Control, Medical Center - University of Freiburg, Faculty of Medicine, University of Freiburg, Freiburg, Germany

^4^ Member of the German Center of Lung Research (DZL), Munich, Germany

^*^ These authors contributed equally to this work

Corresponding author:

Prof. Dr. Francesca Alessandrini,

e-mail: [francesca.alessandrini@tum.de](mailto:francesca.alessandrini@tum.de)

**Preparation of OSB**

OSB was obtained from an industrial mill directly after production. The boards were made of the wood species *Pinus sylvestris* L. and bonded with pMDI (poly-methylene diphenyl diisocyanate). The VOC emission rates of this OSB batch were determined over a period of 28 days from one test specimen using the emission test chamber method in accordance with DIN EN ISO 16000-9:2008 (1). A glass desiccator (23L) with constant conditions (23 °C ± 2°C, relative humidity of 50% ± 5%; air velocity of 0.1 to 0.3 ms^-1^) was used as test chamber (1). OSB was then cut to the size of the mouse cages (28 cm x 14,5 cm), wrapped in aluminum foil and stored at -20°C until the start of the experiment. The boards used in this study (Fig. 1) belonged to the same batch of OSB, whereby lower-emitting boards were stored for one year at -20 °C.

**Mice**

Six week old female C57BL/6J mice (Charles River Laboratories, Sulzfeld, Germany) were kept under controlled humidity, temperature (22 ± 2 °C) and pathogen-free conditions in individually ventilated cages (VentiRack; Biozone, Margate, UK) with a 12 h light-dark cycle. To avoid cage effects, animals from same breeding compartment were randomly assigned to experimental groups before each experiment. All experiments were carried out under federal guidelines for the use and care of laboratory animals and approved by the government of the district of upper Bavaria (Approval n. ROB-55.2-2532.VET_02-16-198).

**Experimental models of AD and exposure to OSB emissions**

To investigate the influence of OSB emissions on the development of AD, two well established models using two different irritants triggering an inflammatory skin disease on mouse ears, a non-antigen specific (calcipotriol, Fig. 2A) and an antigen-specific (oxazolone; Fig. 2B), were chosen. The rationale behind inducing the model on mouse ears instead of on dorsal skin is that inducing the model on mouse ears avoids the procedure of hair removal and allows for measurements of ear thickness as a surrogate marker for skin inflammation (2, 3). The calcipotriol model, described in (4), was adapted from Li et al. (5). In short, calcipotriol (Calcipotriol hydrate, Sigma-Aldrich, St. Louis, USA) was dissolved in 100 % EtOH (w/v) (Merck, Darmstadt, Germany) and 14 µl of a 1.125 nmol solution was topically applied three times a week on both ears. The oxazolone model described in (4) was adapted from Man et al. (6) (Fig. 2B). For this model, oxazolone (4 Ethoxymethylene-2-phenyl-2-oxazolin-5-one) (Sigma-Aldrich) was dissolved in 100 % EtOH (Merck) and 20 µl of a 0.8 % (w/v) solution was topically applied on day 0 on both ears for sensitization. From day 8 on, mice were challenged 3 times a week by topical application of 20 µl of 0.4 % (w/v) oxazolone in 100 % EtOH. For both models, control groups received same amount of 100 % EtOH.

At every treatment time point, body weight, TEWL and ear thickness were recorded as described in (4). Each application of substances on mouse ears and following measurements occurred under a brief 5% sevoflurane (Piramal Critical Care Deutschland GmbH, Munich, Germany) sedation. TEWL was measured on the right ear, using an Aquaflux AF200 (Biox System Ltd, London, UK) and ear thickness was determined using a caliper (Kroeplin; Schlüchtern, Germany) and recorded as the mean of three independent measurements. Skin pH was measured at the beginning (day 0) and at the end of experiment (day end) on the left ear using a skin pH meter and data were analyzed following manufacturer instructions (Courage+Khazaka electronic GmbH, Cologne, Germany) (4).

Higher- or lower-emitting OSB were placed at the bottom of the cages and their edges were covered with a metal frame to avoid mice gnawing at the boards. Vermiculite was used as wood- free bedding (Moosmann GmbH & Co. KG, Ravensburg, Germany) to prevent any confounding emissions. As the decrease of emission rates in the mouse cages occurs faster compared to the one occurring in the absence of continuous ventilation, as in households (7-10), the OSB-samples were replaced weekly to maintain the expected range of TVOC concentrations throughout the duration of the experiment.

**Air sampling and analysis of VOCs**

The VOC concentrations in the cages depend on various factors, mainly on the emission profile of the wooden materials, the product loading factor (ratio of exposed surface area of the OSB and the cage volume) as well as the climatic conditions (air change rate, temperature, humidity) in the cages. Cage air was monitored three times a week on day 1, day 3 and day 7. The air sampling in the cages and the determination of VOC was performed according to DIN ISO 16000-6:2012 (11). For this purpose, stainless steel sampling tubes filled with Tenax® TA sorbent (Markes International; Offenbach am Main, Germany) and spiked with 200 ng toluene-d_8_ as internal standard were connected to the cages and a sampling pump (Analyt-MTC Messtechnik GmbH, Müllheim, Germany) working at constant flow rate of 100 mL/min. Depending on the expected VOC concentration the sampling volume ranges between 0.1 and 1.0 L. Tubes were stored at 4 °C until further analysis by thermal desorption (TD) and gas chromatographic mass spectrometric (GC-MS) analysis. TD was performed by using the Ultra Series 2 50:50 (auto-sampling unit) and the Unity Series 2 (thermodesorption unit), (Markes International Ltd, Bridgend, UK). Individual chemical compounds were separated by using a 7890A gas chromatograph (Agilent Technologies Inc., Santa Clara, USA) and were identified and quantified by quadrupole mass spectrometry (5975C, Agilent Technologies Inc.). A VF1701ms capillary column (CP9151, Agilent Technologies Inc.) was used for separation with helium as carrier gas. The GC temperature program included the following steps: 32 °C hold for 3 min, increase by 6 °C min^-1^ to 90 °C, 90 °C hold for 4 min, increase by 8 °C min^-1^ to 200 °C, increase by 12 °C min^-1^ to 240 °C, 240 °C hold for 2 min. The MS was operated in scan mode with 5 scans s^-1^ in the mass range of 22 and 300 u. For most of the VOCs the compound-specific response factors were determined with a multi-point calibration with reference compounds. Substances without reference compounds were quantified using the response factor of compounds with similar chemical structure or the internal standard. Data were processed using the software MSD ChemStation E.02.00.493 (Agilent Technologies Inc.). TVOCs measured represent the sum of the selected VOCs concentrations in µg/m^3^, namely terpenes (α-pinene, β-pinene, 3-carene, limonene, terpinolene, camphene, p-/o-cymol,), saturated aldehydes (pentanal, hexanal, octanal, nonanal, decanal), unsaturated aldehydes (2-heptenal, 2-octenal), aromatic aldehydes (benzaldehyde) and organic acids (acetic acid, propionic acid, hexanoic acid). This corresponds to ≥ 95 % of the sum of all the detected VOCs. Only a defined number of VOC which significantly characterized the air sampled in the cages is displayed in Fig. 1.

**Analysis of lung inflammatory cell infiltration and serum total IgE**

At the end of experiment, mice were anesthetized intra-peritoneally with Ketamine 10 mg/ml, Xylazine 0.2 mg/ml in sterile isotone saline, 10 μl/g body weight and exsanguinated via the retro-orbital plexus and the vena cava. Bronchoalveolar lavage (BAL) and subsequent analysis of BAL cellular infiltrate were performed as previously described (12). After BAL, lungs right lobes were minced and digested in RPMI medium supplemented with 100 µg/ml DNAse (Sigma-Aldrich) and 1 mg/ml collagenase Type 1A (Sigma-Aldrich) at 37 °C. Cell suspension was filtered through a 70 µm cell strainer and cell filtrate was centrifuged (400 g, 5 min., 4 °C). The pellet was resuspended in 6ml 40 % Percoll in RPMI (v/v), layered onto 80 % Percoll in RPMI (v/v) (GE Healthcare-Life Science, Chicago, IL, USA) and centrifuged (1600 g, 15 min., RT) without brake. Cells were collected from the interface, resuspended in PBS and stained for surface markers. Flow cytometric analysis was performed by using a BD LSRII Fortessa flow cytometer (BD Bioscience) and data were analyzed using FlowJo, version 10 software (Tree Star, Ashland, OR, USA). The gating strategy to identify the different lymphoid populations is presented in Fig. S1. Antibodies used for flow cytometry are listed in Table S1. For the measurement of serum total IgE, 150-200 μl blood was collected at the beginning of the experiment (day 0) via the retro-orbital plexus under a brief 5% sevoflurane sedation and at the end of experiment (day end). The sera were analyzed with LEGENDplex Mouse IgE assay (BioLegend^®^, San Diego, CA, USA) following manufacturer’s recommendations.

**Real time-PCR analysis**

After BAL, lung tissue was snap frozen in liquid nitrogen and disrupted using a Tissue Lyser LT (Qiagen GmbH, Hilden, Germany). RNA was extracted using the RNeasy Mini Kit (Qiagen) according to supplier's instructions and directly reverse-transcribed (RevertAid H Minus First Strand cDNA Synthesis Kit, Thermo Fisher Scientific). Quantitative real-time PCR was performed using SYBR Green PCR Kit Master Mix (Qiagen) and the LightCycler^®^480 System (Roche, Basel, Switzerland) according to the manufacturer’s protocol. Primer sequences are listed in Table S2. The expression levels were normalized to GAPDH house-keeping gene and relative changes were represented as 2^−ΔΔCT^ (ΔΔCT=ΔCT−ΔCTControl) (13).

**Histopathology, immunohistochemistry and immunofluorescence**

After BAL, lung tissue (left lobe) and ear tissue were fixed in 4 % paraformaldehyde in PBS for 48 h at room temperature, processed and embedded in paraffin. Tissue sections (4 µm) were prepared for periodic acid–Schiff (PAS) staining (lungs) and hematoxylin and eosin (skin), according to standard protocols. Histopathological analysis was performed on a Leica DM4 B light microscope (Leica, Wetzlar, Germany). For lung samples, mucus hypersecretion and inflammatory cell infiltrate were graded in a scale from 0 to 4 as previously described (12). For skin samples, the sizes of epidermis and dermis were measured using the LASX software (Leica Application Suite X v. 3.4.2), as previously described (4). For this purpose, ears embedded and cut as vertically as possible were measured by comparable tissue orientation using lines drawn perpendicularly to the epidermal surface. Immunohistochemical staining was performed on 2 µm sections of formalin-fixed paraffin-embedded specimens using an automated staining system (Leica). After dehydration, the slides were pre-treated with epitope retrieval solution (ER)1 (for IBA1) or ER2 (for CD4), incubated with the respective primary antibody (IBA1: Wako 019-1974, Cell Signalling 39197; CD4: clone GHH4, Dianova DIA-404) and detected using the Polymer Refine Detection Kit (Leica). All primary antibodies were detected using a secondary anti-rabbit antibody included in the kit. Only for the primary rat antibody (CD4), a secondary rabbit-anti-rat (Vector laboratories) was added. After counterstaining with hematoxylin, slides were digitalized with an automated scanning system (Leica AT2, Wetzlar, Germany). For immunofluorescence analysis of skin samples, the following primary antibodies were used: rabbit anti-mouse 5-lipoxygenase (a kind gift of Olof Rådmark, Karolinska Institute Stockholm, Sweden, 1:100); polyclonal goat anti-mouse COX-2 (Cayman Chemicals, Ann Arbor, MI, USA, 1:100); rabbit anti-mouse mPGES-1 (a kind gift of Per-Johan Jakobsson, Karolinska Institute Stockholm, Sweden, 1:50); rat anti-mouse F4/80 (eBioscience, Thermo Fisher Scientific, Waltham, MA, USA, 1:50); monoclonal rabbit anti-mouse 15-lipoxygenase (Abcam, Cambridge, UK, 1:50). As secondary antibodies Alexa Fluor 488 conjugated donkey anti-rat IgG, Alexa Fluor 647 conjugated donkey anti-rabbit IgG and Alexa Fluor 568 conjugated donkey anti-goat (Life Technologies, Zug, Switzerland) were used and the nuclei were counterstained with DAPI (Life Technologies) (14). Stained tissue sections were imaged with a Leica TCS SP5 II confocal microscope (Leica Microsystems) with 63x or 20x objectives.

**Exposure of a human *in vitro* model of AD to OSB emissions**

Primary human epidermal keratinocytes were isolated by suction blister from healthy donors.

The study was approved by the local ethical committee of the Klinikum rechts der Isar, Technical University of Munich, Project number 5590/12 and 44/16 S. Cells were cultured in DermalLife basal medium (LifeLine Cell Technology; Carlsbad, USA) supplemented with DermalLife K LifeFactor Kit (Lifeline Cell Technology) in a T75 flask at 37 °C, 5 % CO_2_ until 70% confluency. Cells were then transferred to collagen pre-coated (1% in PBS, collagen type I, Sigma-Aldrich, Taufkirchen, Germany) polycarbonate inserts (Merck Millipore, Burlington, MA, USA) with a density of 0.42 x 10^6^ cells/transwell in 500µl supplemented DermaLife basal medium plus 1.5 mM CaCl_2_ (Carl Roth GmBH, Karlsruhe, Germany) to initiate the 3D culture. Each insert was cultured in a 6 well plate with 1.5 mM CaCl_2_-containing medium. After 24 h, the medium in the transwell was removed to place the model at the air-liquid interface (ALI) while the medium in the lower chamber was further supplemented with 1.5 mM CaCl_2_ and 50 µg/ml Vitamin C (Sigma Aldrich). Prior to a 12 h-long stimulation with AD derived T cell supernatant (diluted 1:2), the 3D models were starved for 12 h in DermalLife Basal Medium without supplements. AD derived T cell supernatant was generated as explained in (15). In brief, AD full skin biopsies (n=3) were cultivated in RPMI 1640 medium supplemented with 5% human serum, 0.1 mM NEAA, 2 mM L-Glutamine, 1 mM sodium pyruvate and 100 U/ml penicillin/streptomycin (all Gibco, Paisley, UK) in presence of 60 U/ml IL-2 (Novartis Pharma, Nürnberg, Germany) at 37 °C and 5% CO_2_. Fresh medium containing 60 U/ml IL-2 was replaced three times a week until lesional T cells emigrated from the biopsy. T cells were expanded by α-CD3 and α-CD28 (BD Bioscience, San Jose, CA, USA) stimulation (each 0.75 μg/ml, α-CD3 pre-coated on plate in PBS, α-CD28 soluble) and finally stimulated for 72 h with α-CD3 and α-CD28 as described above. Cell- free supernatant was obtained, characterized by ELISA for IL-4, IL-13 (both BD Biosciences), IL-17, IL-22, IFNγ, TNF (R&D, Minneapolis, MN, USA) and mixed in equimolar ratio. Before exposure of the skin equivalents to pinewood emissions, the T cell supernatant was removed and replaced with DermalLife basal medium with supplements excluding hydrocortisone. Non stimulated cells were handled the same way omitting only the stimulus of T cells supernatants. Exposure to VOCs was accomplished by means of a VITROCELL® 12/6 CF *in vitro* exposure system for the direct exposure of cell cultures to airborne substances (Vitrocell Systems, Waldkirch, Germany), conducted under ALI conditions (16). The exposure system was encased in a climate chamber (ESPEC PR-4ST, Japan) to ensure physiological conditions (37 °C) and ultimately to prevent the formation of VOC-condensation within the exposure system components. The composition of the VOCs mix used in the exposure atmosphere was put together based on the percentage of TVOCs emitted from a 2978 μg/m^3^-emitting OSB plate, therefore in the range of the lower-emitting OSB. Test atmospheres were prepared using PVDF coated 3 L-gas-sampling bags (ALTEF®, Restek Corp., USA) spiked up with liquid reference compounds to a total mass concentration of 5 mg VOCs/L, according to the data given in Tab. S3. For this purpose, mass equivalent volumes of the most representative VOCs (α-pinene, 3-carene, limonene, hexanal and 2-octenal; Merck), excluding acetic acid, were injected into the gas-sampling bag filled with 3 L of clean air. The single test concentration of 5 mg/L was chosen based on the extrapolation from *in vitro* to *in vivo* scaling factors, the duration of the respective exposures, and the longstanding laboratory experience with the same exposure system (16, 17). Spiked gas sampling bags were allowed to equilibrate and volatilize the liquid VOC- test compounds for 60 min at normal pressure at 37 °C. Gas-phase equilibriums reached in test atmospheres, ultimately representing the effective exposure levels of the VOC-mixture, were estimated semi-quantitatively by means of gas chromatography coupled to a flame ionization detector (GC-FID)-analytics. For this purpose, 1 mL of the VOC gas sample was collected from the gas sampling bag prior to exposure and immediately analysed by GC-FID. Instrument and settings were as follows: Varian CP-3800 with FID-detector at 275 °C, J&W DB-624 GC Column, 30 m, 0.32 mm, 1.80 µm, 7 inch cage (DB-624), injection temperature: 220°C, split ratio: 1:200, flow rate: 1.7 mL/min, column temperature program: 140 °C (0.25 min), 10 °C/min to 220 °C (1 min), carrier gas: N_2_. The vacuum-driven mass flow, from the gas-sampling bag up to the exposure system, was set to 3 mL/min. Cells were exposed for 2 h and post-incubated for additional 22 h in the incubator under ALI conditions (37 °C, 5% CO_2_). The basolateral cell culture supernatant was then harvested and stored at -80 °C until analysis.

**Measurement of inflammatory mediators**

For *in vivo* experiments, ear tissue was pre-cut in tissue buffer (Thermo Scientific, Waltham, MA, USA), sonicated and centrifuged for at 12.000 g for 20 min. at 4 °C; the supernatant was used for analysis of cytokines, chemokines and eicosanoids. Quantification of cytokines and chemokines was performed by using the Th Cytokine panel (13-plex) and the proinflammatory panel (BioLegend^®^) or Mesoscale (Meso Scale Diagnostics, Rockville ML, USA) following manufacturer’s recommendations. Analysis of Leukotriene B_4_ (LTB_4_) or 15(s)-hydroxy Eicosatetraenoic acid (15(S)-HETE) was performed using the respective ELISA Kits (Cayman Chemicals), as previously described (18). For *in vitro* experiments, cell culture supernatants were analyzed for cytokines using a human inflammatory panel 1 (13-plex) LEGENDplex ^TM^ (BioLegend^®^). Concentration of all analytes was determined based on a known standard curve using data analysis software LEGENDPlex TM version v8.0, MSD Discovery Workbench Mesoscale Software or Gen5 2.00 software. Results from *in vivo* experiments were normalized to sample’s protein content measured by BCA assay (Pierce Biotechnology, Waltham, MA, USA).

**References**

1. Standards-committee. Determination of the emission of volatile organic compounds from building products and furnishing - Emission test chamber method (ISO 16000-9:2006); German version EN ISO 16000-9:2006. DIN EN ISO 16000-9:2008-04. Indoor Air 2008; Part 9: 1-26.

2. Naidoo K, Jagot F, van den Elsen L, Pellefigues C, Jones A, Luo H, Johnston K, Painter G, Roediger B, Lee J, Weninger W, Le Gros G, Forbes-Blom E. Eosinophils Determine Dermal Thickening and Water Loss in an MC903 Model of Atopic Dermatitis. J Invest Dermatol 2018;138(12):2606-16.

3. Funding AT, Johansen C, Gaestel M, Bibby BM, Lilleholt LL, Kragballe K, Iversen, L. Reduced oxazolone-induced skin inflammation in MAPKAP kinase 2 knockout mice. J Invest Dermatol 2009;129(4):891-8.

4. Amar Y, Schneider E, Koberle M, Seeholzer T, Musiol S, Holge IM, Gschwendtner S, Krappmann D, Steiger K, Biedermann T, Schmidt-Weber CB, Alessandrini F. Microbial dysbiosis in a mouse model of atopic dermatitis mimics shifts in human microbiome and correlates with the key pro-inflammatory cytokines IL-4, IL-33 and TSLP. J Eur Acad Dermatol Venereol 2022;36(5):705-16.

5. Li M, Hener P, Zhang Z, Kato S, Metzger D, Chambon P. Topical vitamin D3 and low-calcemic analogs induce thymic stromal lymphopoietin in mouse keratinocytes and trigger an atopic dermatitis. Proc Natl Acad Sci U S A 2006;103(31):11736-41.

6. Man MQ, Hatano Y, Lee SH, Man M, Chang S, Feingold KR, Leung DYM, Holleran W, Uchida Y, Elias PM. Characterization of a hapten-induced, murine model with multiple features of atopic dermatitis: structural, immunologic, and biochemical changes following single versus multiple oxazolone challenges. J Invest Dermatol 2008;128(1):79-86.

7. Hernandez G, Wallis SL, Graves I, Narain S, Birchmore R, Berry T-A. The effect of ventilation on volatile organic compounds produced by new furnishings in residential buildings. Atmospheric Environment: X. 2020;6:100069.

8. Fürhapper C, Habla E, Stratev D, Weigl M, Dobianer K. Living Conditions in Timber Houses: Emission Trends and Indoor Air Quality. Front Built Environ 2020;5:1-8.

9. Englund F. Emissions of volatile organic compounds (VOC) from wood. 1999. [*https://www.diva-portal.org/smash/record.jsf?pid=diva2%3A1079819&dswid=6933*](https://www.diva-portal.org/smash/record.jsf?pid=diva2%3A1079819&dswid=6933)

10. Makowski M, Ohlmeyer M, Meier D. Long-term development of VOC emissions from OSB after hot-pressing. Holzforschung 2005;59:519-23.

11. Standards-committee. Determination of volatile organic compounds in indoor and test chamber air by active sampling on Tenax TA® sorbent, thermal desorption and gas chromatography using MS or MS-FID (ISO 16000-6:2011). DIN ISO 16000-6:2012-11. Indoor Air 2012; Part 9: 1-26.

12. Alessandrini F, Schulz H, Takenaka S, Lentner B, Karg E, Behrendt H, Jakob T. Effects of ultrafine carbon particle inhalation on allergic inflammation of the lung. J Allergy Clin Immunol 2006;117(4):824-30.

13. Marzaioli V, Aguilar-Pimentel JA, Weichenmeier I, Luxenhofer G, Wiemann M, Landsiedel R, Wohlleben W, Eiden S, Mempel M, Behrendt H, Schmidt-Weber C, Gutermuth J, Alessandrini F. Surface modifications of silica nanoparticles are crucial for their inert versus proinflammatory and immunomodulatory properties. Int J Nanomedicine 2014;9:2815-32.

14. Esser-von Bieren J, Volpe B, Sutherland DB, Burgi J, Verbeek JS, Marsland BJ, Urban JF, Harris NL. Immune antibodies and helminth products drive CXCR2-dependent macrophage-myofibroblast crosstalk to promote intestinal repair. PLoS Pathog 2015;11(3):e1004778.

15. Lauffer F, Jargosch M, Krause L, Garzorz-Stark N, Franz R, Roenneberg S, Böhner A, Mueller NS, Theis FJ, Schmidt-Weber CB, Biedermann T, Eyerich S, Eyerich K. Type I Immune Response Induces Keratinocyte Necroptosis and Is Associated with Interface Dermatitis. J Invest Dermatol 2018;138(8):1785-94.

16. Gminski R, Tang T, Mersch-Sundermann V. Cytotoxicity and genotoxicity in human lung epithelial A549 cells caused by airborne volatile organic compounds emitted from pine wood and oriented strand boards. Toxicol Lett 2010;196(1):33-41.

17. Albrecht W. Which concentrations are optimal for in vitro testing? EXCLI J 2020;19:1172-3.

18. Henkel FDR, Friedl A, Haid M, Thomas D, Bouchery T, Haimerl P, de Los Reyes Jiménez M, Alessandrini F, Schmidt-Weber CB, Harris NL, Adamski J, Esser-von Bieren J. House dust mite drives proinflammatory eicosanoid reprogramming and macrophage effector functions. Allergy 2019;74(6):1090-101.
